# Determinants of fertility issues experienced by young women diagnosed with breast or gynaecological cancer – a quantitative, cross-cultural study

**DOI:** 10.1186/s12885-018-4766-y

**Published:** 2018-09-06

**Authors:** Aleksandra Sobota, Gozde Ozakinci

**Affiliations:** 1 School of Medicine, University of St Andrews, KY16 9TF St Andrews, Scotland, UK; 2 Coombe Women and Infants University Hospital, 8 Cork Street, Merchants Quay, Dublin, D08 XW7X Ireland

**Keywords:** Cancer, Fertility, Oncofertility, AYA cancer, Gynaecological cancer, Breast cancer, Quality of life, Cross-cultural

## Abstract

**Background:**

Although there is a recognition of the importance of fertility to young women with cancer, we do not know who is at risk of distress related to fertility issues following diagnosis. We investigated the determinants of fertility-related distress adopting a cross-cultural perspective and using the Common Sense Model (CSM). We chose the CSM as a theoretical framework as it allows to explore how individuals conceptualise illness within the socio-cultural context.

**Methods:**

British and Polish women with breast or gynaecological cancer were recruited through outpatient clinics or online outlets and completed a questionnaire. Linear regression, mediation and moderated mediation methods were performed.

**Results:**

One hundred sixty-four women participated (mean age 34.55 (SD = 6.66); 78.7% had gynaecological cancer). The determinants of fertility-related distress were: country of origin, recruitment site, negative affect, desire to have children, treatment regret, and total illness perception score. The impact of the desire to have children on fertility-related distress was mediated by psychological value of children, perceived consequences of cancer on one’s life, emotional representation, and treatment-related regret. Country of origin moderated the relationship between the desire to have children and fertility-related distress when mediated by treatment-related regret.

**Conclusions:**

The CSM proved useful in investigating predictors of fertility-related distress, with emotional, rather than cognitive representation of illness determining its levels. Socio-cultural background played a role in determining one’s fertility-related distress and contributed to the explanation of the relationship between one’s desire to have children, treatment-related regret, and fertility-related distress.

**Electronic supplementary material:**

The online version of this article (10.1186/s12885-018-4766-y) contains supplementary material, which is available to authorized users.

## Background

Progress in screening programmes and cancer treatment means that an increasing number of individuals survive cancer diagnosis [1]. Hence, improving quality of life in the survivorship period becomes an important challenge for healthcare professionals.

While the prevalence of cancer in young people (aged 15–49) remains relatively low [2], evidence suggests that when diagnosed, young patients present with distinctive, age-specific information and supportive care needs [3–6]. These include the need for age-appropriate cancer information and state-of-the-art treatments that fit within young people’s lifestyles, information about health behaviours, complementary, and alternative therapies, information about access to peer support, as well as fertility-related needs such as the desire to be provided with information about the impact of cancer treatments on fertility and counselling regarding fertility issues encountered after the end of treatment [7–9]. While the majority of young cancer patients express these needs [7] and their importance has been recognised by the leading practice guidelines [10–12], they often remain unmet by the healthcare providers [7].

Fertility-related issues are expressed by both men and women with cancer, however, the burden of reproductive concerns affects female cancer patients in particular. This could be due to the fact that while there are fertility preservation methods available to women diagnosed with cancer, including egg or embryo storage, these are more, personally engaging, time consuming, and costly than sperm storage accessible to men.

Several reviews have investigated various psychological aspects of oncofertility as they pertain to women [7–9, 13–16]. While some examine female cancer patients’ knowledge [8], information needs [7, 9], and preferences regarding discussions about fertility [8], others contribute to our understanding of the meaning of fertility to women with cancer [15], how it changes over time [14] and how fertility issues related to cancer treatment can affect women’s psychosocial outcomes [13, 14, 16]. There is, however, a paucity of evidence helpful in determining who might be affected by distress related to fertility issues post-cancer, with four available studies [17–20] pointing towards relationship and childbearing status as well as the desire to have children as being potential risk factors.

There are also two main shortcomings of the current literature. The first one is the cultural homogeneity of the existing studies [16]. Not only were the majority of the studies conducted in a very limited number of locations including the US, Australia, and Western Europe, but also their samples consisted mainly of well-educated, predominantly while women with relatively high income [16]. However, as suggested by Greil et al. [21], the concept of fertility is embedded in the broader socio-cultural context, and therefore infertility would not necessarily have the same psychological effect on women with different socio-cultural backgrounds. Indeed, a multi-national study exploring women’s attitudes towards cancer treatment carrying a potential threat to affect reproductive outcomes [22] showed that young breast cancer patients differed in terms of the risk to their fertility they were willing to accept based on their country of origin. Specifically, women from Western Europe were more likely to accept the risk of infertility resulting from chemotherapy than their counterparts from Eastern Europe, South Africa, Middle East, or South America [22].

The second limitation of the literature is the lack of theoretical underpinnings of the existing studies. Whilst this does not preclude studies from producing valid and meaningful results, the lack of theoretical framework may prevent the researchers from uncovering important concepts or processes, and affect their explanations of the findings [23].

To address the aforementioned gaps in the existing evidence, this study aimed to determine the predictors of distress related to reproductive issues in young gynaecological and breast cancer survivors (diagnosed at the age between 18 and 45), accounting for the potential impact of socio-cultural background and using a theoretical framework of the Common Sense Model (CSM) [24–26].

The CSM arises primarily from the psychological concept of self-regulation and explains how people react to illness. It assumes that a health threat elicits two simultaneous responses in an individual – an emotional (such as fear or distress) and a cognitive response (a representation of threat) which in turn promote coping strategies to manage both the emotions and the threat. Those two responses are defined by illness perceptions which refer to the nature of a health threat and are conceptualised according to the following categories:

1. *Identity* that includes the label of an illness [its name (e.g., diabetes)] and the associated symptoms.
2. *Timeline* that represents different time frameworks relating to an illness such as the time needed to diagnose it, its duration, or time needed for recovery.
3. *Causes* that can be classified as intrinsic or extrinsic to an individual or as environmental, biological, emotional or psychological [27] Causal attributions differ depending on the disease stage they are made at (e.g., attribution about a symptom; attribution about a disease) [28].
4. *Consequences* that refer to the seriousness of an illness and its influence on different life domains.
5. *Cure/control* that represents the degree to which an individual has control over an illness and assesses it as curable [29].

However, the usefulness of the CSM relies not only on the fact that it explains how individuals conceptualise and attach meaning to their illness by understanding their health condition through illness perceptions, but also on the fact that these processes do not occur in a vacuum but in a particular socio-cultural context.

To investigate the latter, we chose to concentrate on women from two countries – the UK as an example of a Western European country and Poland as an example of an Eastern European country. While we could expect differences in terms of fertility-related distress between these two populations as purported by Senkus et al. [22], we wanted to verify whether these results could be replicated and if so, examine more in depth the reason why these differences occurred. Another, more practical rationale to choose these two particular populations stemmed from the fact that Polish ethnic group has grown to become one of the largest minorities in the UK [30], and therefore any available evidence could contribute to the improvement in the delivery of healthcare services to Polish women who live in the UK.

## Methods

We conducted a multi-centre cross-sectional survey. Reproductive age women diagnosed with gynaecological or breast cancer were invited to participate in the study (for details of inclusion criteria see Additional file 1: Table S1). They were approached through several outlets including oncology outpatient clinics in Scotland and Poland, Scottish Health Research Register (SHARE), as well as online outlets of the UK-based and Polish cancer charities and support organisations. The questionnaire was available in a paper or online version for participant’s convenience. Potential participants were approached and informed about the study in person in the outpatient clinics. The online outlets included websites and social-media profiles of cancer charities and support organisations where a link to the participant information sheet and questionnaire was made available to potential participants. While it was not possible to calculate the overall participation rate for the study due to missing information about the number of research packs accessed out in the outpatient clinic setting, the details of recruitment are presented in Table 1. No compensation was offered for participation in the study.

**Table 1:** Study participation rates by recruitment site

Recruitment site	Questionnaire accessed	Questionnaire returned	Participation rate
Scottish clinics	153	39	25.5%
Polish clinics	unrecorded	36	–
SHARE	13	3	23%
UK-based online outlets	86	77	89.5%
Polish online outlets	17	7	41.2%
Other	unrecorded	2	–

### Measures

The questionnaire (in English and Polish) included the following outcome and predictor variables.

### Outcome variable

Distress related to reproductive issues was measured using the Impact of Event Scale Revised (IES-R) [31, 32]. We used the version adapted to evaluate the impact of fertility impairment on one’s psychological well-being [17]. It consists of 22 items scored on a 5-point Likert scale (with responses ranging from ‘not at all’ to ‘extremely’). The items form three subscales: intrusion, avoidance, and hyperarousal. The subscale scores are produced by obtaining the mean score of the items belonging to a subscale and are within the range of 0–4. The overall scale score is obtained by summing all responses with a range 0–88.

### Predictor variables

The questionnaire measured the following predictor variables: sociodemographic and cancer characteristics, illness perceptions, regret related to the outcome of treatment process (decision regret), trait negative affect, social disapproval of not having children, value of children, and desire to have children at the time of diagnosis. Recruitment site (other vs. online) was determined based on the type of questionnaire a participant completed (paper-based or online) and was also included in the analysis.

Socio-demographic variables included age, country of origin, relationship status, childbearing status, monthly income level before tax, and the highest education level. Disease characteristics included type of cancer diagnosis, stage of cancer at diagnosis, type of treatment received, and date of diagnosis.

Illness perceptions were measured using the 9-item Brief Illness Perception Questionnaire (Brief-IPQ) [33] which uses an 11-point Likert scale to assess eight illness perception dimensions. Two items – concern and emotions – reflect the emotional representation of illness. Concern indicates the degree of concern one has about their illness, while emotions represents the effect of illness on one’s psychological well-being. Five items reflect the cognitive representation of illness and these include consequences, treatment control, personal control, identity, and timeline. Consequences capture the extent to which illness affects one’s life. Treatment control represents the extent to which one believes treatment can help the illness, while personal control measures one’s own perception of control over the illness. Identity indicates the level of burden one experiences due to the symptoms of illness. Timeline probes one’s beliefs about the length of the illness. Finally one item reflects the extent to which one the understand their illness (coherence). The overall score of the scale represents the degree to which an illness is perceived as threatening. While the scale questions were not re-worded to ask about cancer perceptions, women were asked to complete the scale in the context of their cancer diagnosis and treatment.

Regret related to the outcome of the treatment process (decision regret), specifically the fertility impairment due to treatment, was measured using a single item designed for the purpose of the study (‘I regret having undergone treatment that altered my fertility’) with response on a 5-point Likert scale ranging from ‘not at all’ to ‘extremely’. While this question was not piloted prior to its use in the study, it was reviewed by a member of our target patient population for acceptability.

Trait negative affect (NA) was measured using the NA subscale of the Positive and Negative Affect Scale (PANAS) [34, 35] where 10 adjectives (distressed, upset, guilty, scared, hostile, irritable, ashamed, nervous, jittery, and afraid) are rated on a 5-point Likert scale. The total subscale score is produced by summing all responses (range 10–50) with higher score indicating higher level of trait NA (‘To what extent you generally feel this way’).

Two factors related to socio-cultural background potentially impacting the psychological well-being of women with fertility problems were included. First, we assessed the value that women attach to having children using a scale adapted from a cross-cultural study on the value of children (VOC) [36]. The scale consists of 27 items scored on a 5-point Likert scale which form three general dimensions of VOC: the psychological-emotional value (12 items), the economic-utilitarian value (five items), and the social-normative VOC (seven items). Although this last dimension places childbearing in the social context, it does not capture the societal attitudes towards childlessness. This seemed important in view of the research suggesting that living in a society condemning childlessness can contribute to a worse psychological well-being of people who do not have children [37]. Therefore, we measured social disapproval of not having children as a second factor potentially contributing to the psychological well-being of women with fertility issues following cancer diagnosis and treatment. It was evaluated with a question designed for the purpose of the study (‘How much do you think the culture you come from disapproves of people who do not have children?’) using a 5-point Likert scale with responses ranging from ‘not at all’ to ‘extremely’.

Desire to have children at the time of cancer diagnosis was measured by asking participants to evaluate one’s own and partner’s desire to have children on a 5-point Likert scale with responses ranging from ‘not at all’ to ‘extremely’. Participants who were single at diagnosis were asked to rate their own desire to have children only.

### Statistical analysis

Statistical analyses were conducted using Statistical Package for Social Sciences for Windows, version 22 [38]. Additional PROCESS macro was used to conduct mediation and moderation analyses [39].

Descriptive statistics were used to characterize the study population and are reported as frequencies and percentages for categorical variables, medians and ranges for continuous, non-normally distributed variables, and means and standard deviations for continuous, normally distributed variables. Linear regression was used to address the main aim of the study. First, the univariate associations between potential predictors and outcome were investigated using:

1. Parametric tests (*t*-test or one-way ANOVA) where predictor was categorical or,
2. Spearman correlations where predictor was continuous.

Additionally, scatterplots were visually inspected to ascertain the linear relationship between predictors and outcome variables.

Due to a relatively small sample size (*n* = 164), a limited number of predictors (k = 14, calculated based on a formula by Green [40]) could be entered simultaneously into the multivariate regression models. Therefore, only predictors associated with the outcomes at *p* ≤ 0.05 were entered into the final multivariate models.

Hierarchical regression was used to produce multivariable model with control variables entered in the first step, followed by literature-based predictors entered in the subsequent steps and new predictors specific to this study entered in the final step [41]. To avoid multicollinearity, bivariate correlations between predictors were investigated. Where these were higher than 0.8 [41, 42], a decision regarding the exclusion of one of the variables from the model was made on a case by case basis. The final models were tested as to whether they met the regression assumptions as outlined by Field [41].

Further exploratory mediation and moderation analyses were conducted to examine the relationships between the predictor variables and distress related to fertility in more depth. Mediation analyses aimed to examine whether the impact of the predictor ‘desire to have children’ on total distress related to fertility was mediated by other variables included in the original model. The associations between the predictors from the original model and ‘desire to have children’ were explored using Spearman correlations. Where these reached the significance level of *p* ≤ 0.05, they were further tested as mediators. Due to the restriction of PROCESS macro in using dichotomous variables as mediators, treatment-related regret and cultural disapproval of childlessness were used as continuous variables (as opposed to their dichotomised versions used in the regression model).

To further explore cross-cultural differences in the distress related to fertility, the variable ‘country of origin’ was treated as a potential moderator in the mediation models. Correlations among the variables which proved to be significant mediators in the previous analysis (*p* ≤ 0.05), ‘desire to have children’, and ‘distress related to fertility’ were tested in the study sample split by the country of origin. Where differences between the direction or significance level of the associations were detected, moderation analysis was applied to investigate which of the paths of the mediation model were subject to moderation.

The effects and confidence intervals for all the models were calculated for 1000 bias-corrected bootstrapped samples and the significance of the effects was assessed by investigating the bootstrapped 95% confidence intervals.

## Results

A total of 164 women were recruited for the study (See Table 2).

**Table 2:** Sample characteristics

Variable			
	Range	Mean ± SD	
Age at diagnosis (years) (*n* = 148)	19–46	34.55 ± 6.66	
Age at enrolment (years) (*n* = 157)	21–54	37.55 ± 6.87	
Time since diagnosis (years) (*n* = 155)	0–18	3.36 ± 2.93	
	Value	N	%
Country of origin	Britain	118	72.0
	Poland	43	26.2
	Other	2	1.2
	Missing	1	0.6
Partnership status at enrolment	Partnered	122	74.4
	Unpartnered	40	24.4
	Missing	2	1.2
Childbearing status	No children	80	48.8
	1 child	33	20.1
	2 children	35	21.3
	3 or more children	14	8.5
	Missing	2	1.2
Education	Less than university education	66	40.2
	At least some university education	97	59.1
	Missing	1	0.6
Income	Less than average for the country	102	62.2
	More than average for the country	43	26.2
	Prefer not to say	17	10.4
	Missing	2	1.2
Cancer diagnosis	Cervical	58	35.4
	Ovarian	41	25.0
	Uterine	27	16.5
	Other gynaecological	3	1.8
	Breast	35	21.3
Stage of cancer	1	69	42.1
	2	49	29.9
	3	30	18.3
	4	2	1.2
	Missing	14	8.5
Surgery – gynaecological	Conservative	29	22.5
	Radical	82	63.6
	None	18	14
Surgery – breast	Conservative	13	37.1
	Radical	19	54.3
	None	3	8.6
Chemotherapy	Yes	92	56.1
	No	72	43.9
Radiotherapy	Yes	66	40.2
	No	98	59.8
Endocrine therapy	Yes	25	15.2
	No	10	6.1
	N/A	129	78.7

There were no significant differences with regard to any of the socio-demographic or cancer characteristics between the participants recruited in the UK and in Poland. While women recruited online and those recruited via other outlets did not differ in terms of disease characteristics, those recruited online were significantly younger both at the time of enrolment and the time of the diagnosis than those recruited via other outlets (*t* = − 2.87, *p* < 0.01 and *t* = − 2.66, *p* < 0.01, respectively). Women recruited online were also more likely to be in a partnered relationship at the time of enrolment (83.33% vs 66.67%, *p* ≤ 0.05), to not have any children (59.52% vs 38.46%, *p* < 0.01) and to have at least some university education (67.47% vs 51.25%, *p* ≤ 0.05). Also, women with breast cancer were significantly older than women with gynaecological cancer both at the time of enrolment and diagnosis (*t* = − 3.26, *p* < 0.01 and *t* = − 2.96, *p* < 0.01, respectively), and they were less likely to choose the answer ‘prefer not to say’ when asked about their average monthly income (0 vs. 13.39%, *p* ≤ 0.05). To account for these differences, the method of recruitment and the type of cancer diagnosis were controlled for in the subsequent analyses.

### Predicting fertility-related distress

Women’s average level of distress related to fertility issues was 29.36 (*n* = 157, SD = 21.71, range 0–86). Fourteen out of twenty predictor variables were significantly associated with the outcome variable in univariate analyses. Thirteen were entered into the final model (with one – partner’s desire to have children – excluded due to multicollinearity) in the following order which was based on the existing evidence [17–20]:

- Block 1 (control variables): age at diagnosis, country of origin, type of cancer, type of treatment, recruitment site, childbearing status, negative affect
- Block 2: desire to have children
- Block 3: treatment-related regret
- Block 4 (culture-related variables): cultural disapproval of childlessness, utilitarian, social, and psychological VOC
- Block 5: total illness perception score

The model explained 54.8% of the variability in total fertility-related distress and six predictors including the country of origin, recruitment site, negative affect, desire to have children, treatment regret, and total illness perception score remained individually significant (See Table 3). Excluding control variables, treatment-related regret and illness perception were the two strongest predictors of total fertility-related distress (β = 0.16, *p* ≤ 0.05 and β = 0.23, *p* < 0.01, respectively).

**Table 3:** Multivariate model predicting total fertility-related distress

	B	SE B	β	*p*
Step 1 – control variables				
Constant	25.49	10.37		*≤0.05*
Age at diagnosis	−0.45	0.25	−0.14	*n.s.*
Country of origin (Britain vs Poland)	12.45	3.67	0.24	*< 0.01*
Type of cancer (gynaecological vs breast)	3.14	5.01	0.059	*n.s.*
Type of treatment (sterile vs uncertain fertility)	−9.62	4.16	− 0.22	*≤0.05*
Recruitment site (other vs online)	−10.42	3.20	−0.24	*< 0.01*
Childbearing status (no vs yes)	−3.24	2.90	−0.075	*n.s.*
Negative affect	1.06	0.153	0.461	*< 0.01*
Step 2 – desire to have children				
Constant	3.61	11.71		*n.s.*
Age at diagnosis	−0.17	0.25	−0.053	*n.s.*
Country of origin (Britain vs Poland)	13.28	3.53	0.271	*< 0.01*
Type of cancer (gynaecological vs breast)	2.32	4.82	0.044	*n.s.*
Type of treatment (sterile vs uncertain fertility)	−8.18	4.01	−0.184	*≤0.05*
Recruitment site (other vs online)	−9.27	3.09	−0.214	*≤0.05*
Childbearing status (no vs yes)	−0.48	2.89	−0.011	*n.s.*
Negative affect	1.06	0.15	0.46	*< 0.01*
Desire to have children	3.43	0.97	0.25	*< 0.01*
Step 3 – treatment-related regret				
Constant	−0.41	11.49		*n.s.*
Age at diagnosis	−0.14	0.24	−0.04	*n.s.*
Country of origin (Britain vs Poland)	13.82	3.44	0.28	*< 0.01*
Type of cancer (gynaecological vs breast)	1.45	4.70	0.03	*n.s.*
Type of treatment (sterile vs uncertain fertility)	−5.76	4.00	−0.13	*n.s.*
Recruitment site (other vs online)	−8.18	3.03	−0.19	*≤0.05*
Childbearing status (no vs yes)	0.63	2.84	0.02	*n.s.*
Negative affect	1.03	0.14	0.45	*< 0.01*
Desire to have children	2.97	0.96	0.22	*< 0.01*
Treatment related regret (no vs all others)	8.65	3.00	0.19	*≤0.05*
Step 4 – culture-related variables				
Constant	−6.25	11.80		*n.s.*
Age at diagnosis	−0.22	0.25	−0.07	*n.s.*
Country of origin (Britain vs Poland)	12.21	3.67	0.25	*< 0.01*
Type of cancer (gynaecological vs breast)	2.21	4.71	0.04	*n.s.*
Type of treatment (sterile vs uncertain fertility)	−7.26	4.07	−0.16	*n.s.*
Recruitment site (other vs online)	−8.31	3.05	−0.19	*≤0.05*
Childbearing status (no vs yes)	−0.80	3.07	−0.02	*n.s.*
Negative affect	0.94	0.15	0.41	*< 0.01*
Desire to have children	2.30	1.02	0.17	*≤0.05*
Treatment related regret (no vs all others)	8.27	3.05	0.18	*≤0.05*
Cultural disapproval of childlessness (no vs all others)	3.85	2.74	0.09	*n.s.*
VOC_U	1.50	2.66	0.05	*n.s.*
VOC_S	−0.99	3.01	−0.03	*n.s.*
VOC_P	3.28	2.26	0.12	*n.s.*
Step 5 – illness perceptions				
Constant	−11.68	11.54		*n.s.*
Age at diagnosis	−0.27	0.24	−0.08	*n.s.*
Country of origin (Britain vs Poland)	11.58	3.55	0.24	*< 0.01*
Type of cancer (gynaecological vs breast)	−0.17	4.61	−0.01	*n.s.*
Type of treatment (sterile vs uncertain fertility)	−5.49	3.98	−0.12	*n.s.*
Recruitment site (other vs online)	−6.41	3.01	−0.15	*≤0.05*
Childbearing status (no vs yes)	−2.31	3.01	−0.05	*n.s.*
Negative affect	0.70	0.16	0.31	*< 0.01*
Desire to have children	2.03	0.99	0.15	*≤0.05*
Treatment related regret (no vs all others)	7.27	2.97	0.16	*≤0.05*
Cultural disapproval of childlessness (no vs all others)	2.94	2.66	0.07	*n.s.*
VOC_U	1.28	2.57	0.05	*n.s.*
VOC_S	−1.70	2.92	−0.06	*n.s.*
VOC_P	4.10	2.20	0.15	*n.s.*
Brief-IPQ total	0.35	0.11	0.23	*< 0.01*

Step 1: *R*^2^ = 0.466, adjusted *R*^2^ = 0.438, *F*(7, 134) = 16.69, *p* < 0.01

Step 2: *R*^2^ = 0.512, adjusted *R*^2^ = 0.482, *F*(1, 133) = 12.55, *p* < 0.01, Δ *R*^2^ = 0.046

Step 3: *R*^2^ = 0.541, adjusted *R*^2^ = 0.509, *F*(1, 132) = 8.31, *p* < 0.01, Δ *R*^2^ = 0.029

Step 4: *R*^2^ = 0.561, adjusted *R*^2^ = 0.516, *F*(4, 128) = 1.48, *p* = n.s*.*, Δ *R*^2^ = 0.020

Step 5: *R*^2^ = 0.593, adjusted *R*^2^ = 0.548, *F*(1, 127) = 9.99, *p* < 0.01, Δ *R*^2^ = 0.032

Additional models to investigate specifically which illness perceptions contributed to total fertility-related distress revealed that when entered in the last step, consequences, identity, illness concern, and emotional representation explained the variability in the fertility-related distress (see Additional file 2: Tables S2 to S7). Models including illness concern and emotional representation as final predictors achieved a better fit to the data (adjusted *R*^2^ of 55.2% and 55.6%, respectively) than the model including the total illness perception score.

### Mediation analysis

Initial correlation coefficients revealed five variables which correlated significantly with both ‘desire to have children’ and ‘fertility-related distress’. These included ‘treatment-related regret’, ‘psychological VOC’, and three dimensions of illness perceptions – ‘consequences’, ‘coherence’, and ‘emotional representation’, and were subject to mediation analysis. From these five simple mediation models, ‘desire to have children’ indirectly influenced fertility-related distress separately through treatment-related regret, psychological VOC, consequences, and emotional representation (see Additional file 3: Tables S8-S11 and Figures S1-S4)

A multiple mediation analysis including all four independently significant mediators was performed to investigate whether the mediators remained statistically significant in the presence of others and, if so, whether indirect effects were significantly different from each other.

**Fig. 1:**
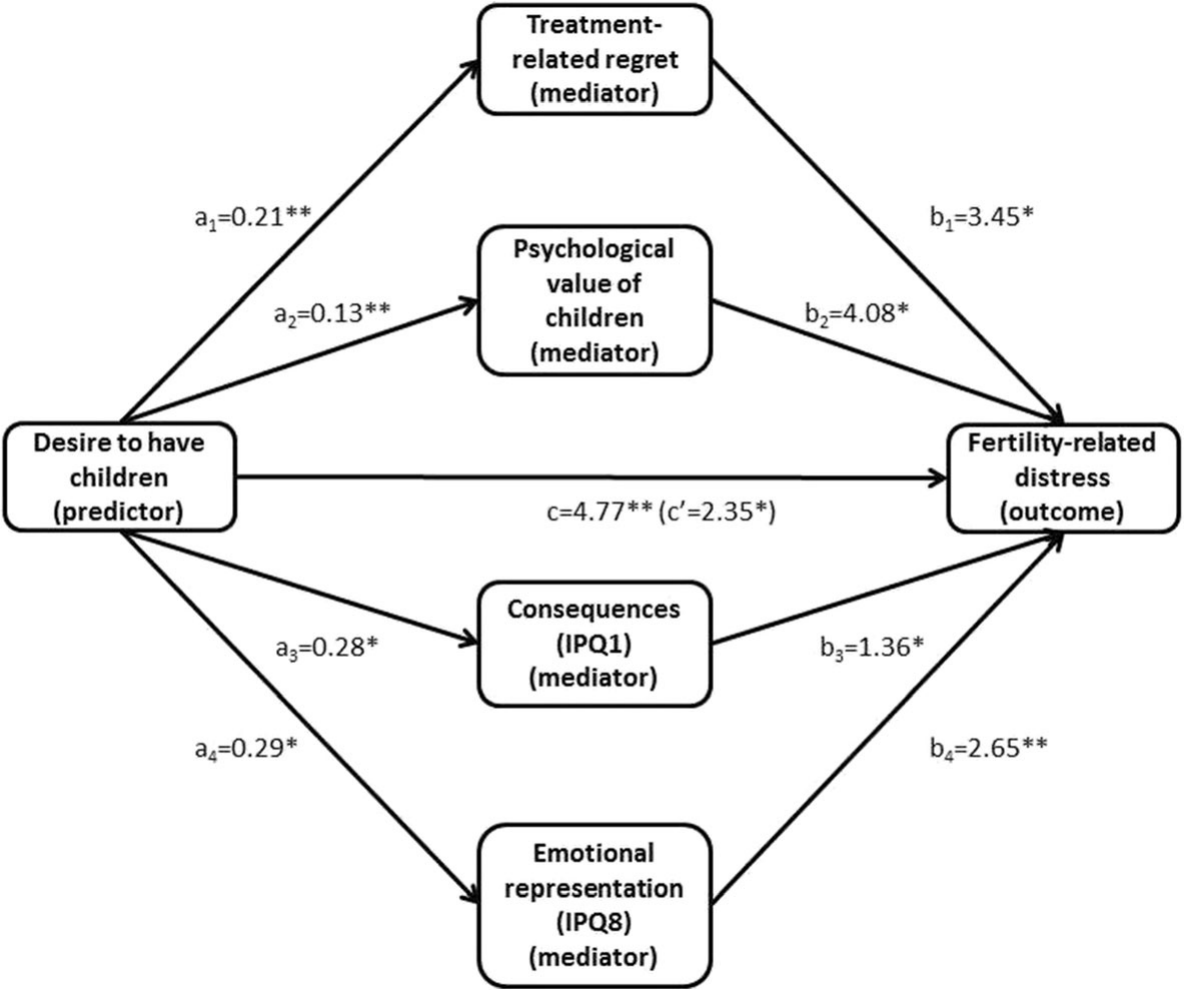
Multiple mediation model with desire to have children as predictor, treatment-related regret, psychological VOC, consequences and emotional representation as mediators and fertility-related distress as outcome (*p ≤ 0.05, **p < 0.01)

As illustrated in Fig. 1, components a and b of all four indirect pathways remained significant in the multiple mediation model (more details available in Additional file 4: Table S12). The investigation of the bootstrapped confidence intervals confirmed that all four indirect effects: a_1_b_1_, a_2_b_2_, a_3_b_3_, and a_4_b_4_ were significant (see Table 4). Pairwise comparisons between the indirect effects (through the investigation of the bootstrapped 95% confidence intervals of contrasts between them) suggested that these effects were not significantly different from each other.

**Table 4:** Multiple mediation model with desire to have children as predictor, treatment-related regret, psychological VOC, consequences and emotional representation as mediators and fertility-related distress as outcome

	Consequent																			
		Treatment-related regret				Psychological VOC				Consequences (IPQ1)				Emotional representation (IPQ8)				Fertility-related distress		
Antecedent		B	SE	*p*		B	SE	*p*		B	SE	*p*		B	SE	*p*		B	SE	*p*
Constant	i_M1_	1.04	0.18	*< 0.01*	i_M2_	3.02	0.13	*< 0.01*	i_M3_	4.76	0.46	*< 0.01*	i_M4_	5.74	0.46	*< 0.01*	i_Y_	−20.98	6.83	*< 0.01*
Desire to have children	a_1_	0.21	0.06	*< 0.01*	a_2_	0.13	0.04	*< 0.01*	a_3_	0.28	0.14	*≤0.05*	a_4_	0.29	0.14	*≤0.05*	c’	2.35	0.97	*≤0.05*
Treatment-related regret		–	–	–		–	–	–		–	–	–		–	–	–	b_1_	3.45	1.37	*≤0.05*
VOC_P		–	–	–		–	–	–		–	–	–		–	–	–	b_2_	4.08	1.83	*≤0.05*
Consequences (IPQ1)		–	–	–		–	–	–		–	–	–		–	–	–	b_3_	1.36	0.65	*≤0.05*
Emotional representation (IPQ8)		–	–	–		–	–	–		–	–	–		–	–	–	b_4_	2.65	0.64	*< 0.01*
Model fit		*R*^2^ = 0.09 *F*(1, 149) = 15.49, *p < 0.01*				*R*^2^ = 0.07 *F*(1, 149) = 10.70, *p < 0.01*				*R*^2^ = 0.03 *F*(1, 149) = 4.21, *p ≤ 0.05*				*R*^2^ = 0.03 *F*(1, 149) = 4.21, *p ≤ 0.05*				*R*^2^ = 0.40 *F*(5, 145) = 19.71, *p < 0.01*		

### Moderation analysis

Associations between treatment-related regret and distress related to fertility, and treatment-related regret and desire to have children differed between the British and Polish participants (see Additional file 5: Tables S13 and S14). Country of origin was therefore investigated as a moderator of those associations.

The first moderation analysis investigating the country of origin as a moderator of the relationship between treatment-related regret and fertility-related distress, showed that while the model was statistically significant (*R*^2^ = 0.16, *F*(3,149) = 9.11, *p* < 0.01), the interaction term between treatment-related regret and country of origin was not.

In the second analysis, examining the country of origin as a moderator of the association between treatment-related regret and desire to have children, the obtained model was statistically significant (*R*^2^ = 0.13, *F*(3,152) = 7.10, *p* < 0.01), as was the interaction term between desire to have children and country of origin (B = − 0.27, *p* ≤ 0.05) (see Additional file 5: Figure S5).

**Fig. 2:**
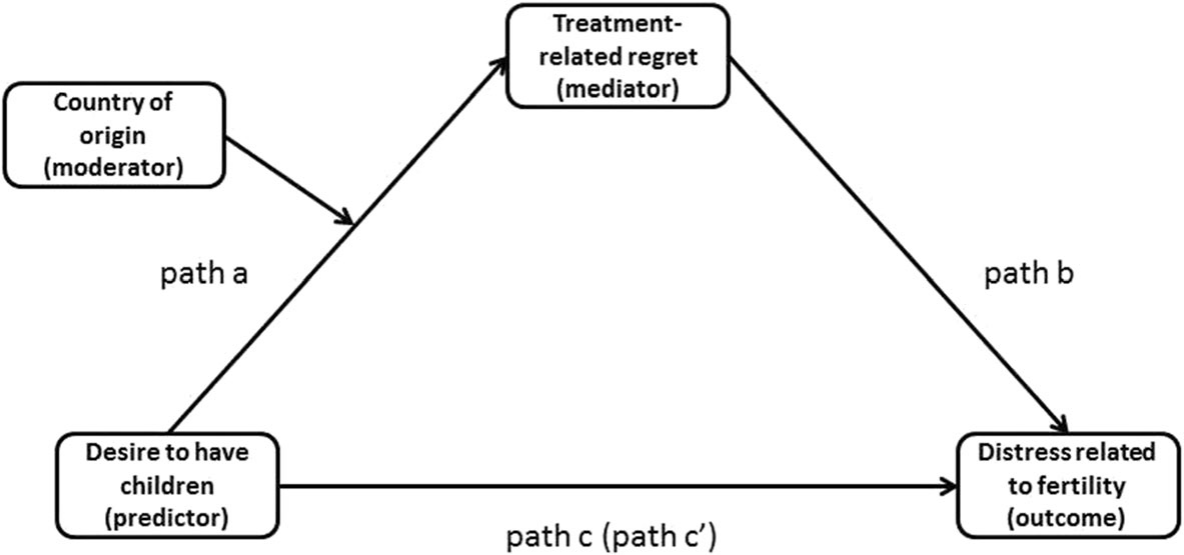
Moderated mediation with the moderator influencing path a

Since country of origin acted as a moderator affecting the relationship between desire to have children and treatment-related regret, it was introduced into the mediation model as shown in Fig. 2.

Country of origin moderated path ‘a’ and hence the indirect path between desire to have children and fertility-related distress through ‘treatment-related regret’ (see Table 5).

**Table 5:** Moderated mediation with desire to have children as predictor, treatment-related regret as mediator, fertility-related distress as outcome and country of origin as mediator influencing path a

		Consequent						
		Treatment-related regret				Fertility-related distress		
Antecedent		B	SE	*p*		B	SE	*p*
Constant	i	0.87	0.15	*< 0.01*	i	11.15	3.78	*< 0.01*
Desire to have children	a_1_	0.28	0.06	*< 0.01*	c’	3.72	1.10	*< 0.01*
Treatment-related regret		–	–	–	b	4.96	1.62	*< 0.01*
Country of origin	a_2_	0.68	0.38	*n.s.*		–	–	*–*
Desire to have children x country of origin	a_3_	−0.27	0.13	*≤0.05*		–	–	*–*
		*R*^2^ = 0.12 *F*(3, 147) = 6.19, *p < 0.01*				*R*^2^ = 0.17 *F*(2, 148) = 15.29, *p < 0.01*		

The unstandardised indirect effect of ‘desire to have children’ on fertility-related distress conditional on country of origin was 1.37 for the British population with the bias-corrected bootstrapped 95% confidence interval entirely above 0 (0.60, 2.52), indicating significance. For the Polish population the indirect effect was 0.008 with the bias-corrected bootstrapped 95% confidence interval including 0 (− 1.13, 1.03), indicating that the effect was not significant (for visual representation of effects see Additional file 5: Figure S6).

The difference between the conditional indirect effects between the British and Polish participants was statistically significant as suggested by the index of moderated mediation which equaled − 1.36 with bias-corrected bootstrapped 95% confidence interval entirely below 0 (− 3.13, − 0.26). These results indicate that while the mediation model held for the British subsample of the participants, it became non-significant for the Polish subsample.

## Discussion

This study sought to determine potential predictors of fertility-related distress among young gynaecological and breast cancer patients, focusing specifically on the role of one’s socio-cultural background and using the CSM as a theoretical framework to guide the analysis and interpretation of the results.

Findings suggest that Polish participants; those recruited through clinics as opposed to online; those with higher desire to have children; those with higher negative affect; those who reported regret with respect to the treatment outcome; and finally those who perceived their illness as more threatening were more likely to experience higher levels of fertility-related distress. An in-depth analysis of illness perceptions also indicates that it was the emotional representation (reflected through more concern with regard to illness and more emotional consequences of the disease), as opposed to the cognitive representation that mainly contributed to higher levels of fertility-related distress. Furthermore, exploratory analyses propose that while desire to have children affects fertility-related distress directly, it can also have an indirect effect through influencing treatment-related regret, psychological VOC, consequences, and emotional representation. Finally, the model investigating the indirect effect of desire to have children through treatment-related regret seems to suggest that this relationship can be affected by one’s socio-cultural background.

This study corroborates the evidence that the wish to have a child or more children is a likely predictor of post-treatment distress related to fertility among young women with cancer. However, it does not support the evidence suggesting that not having children or being single may contribute to higher distress. This is potentially important for clinical practice in that a patient’s preferences regarding family life, rather than objective indicators such as relationship or childbearing status, seem to determine the patient’s emotional responses post-treatment. These findings emphasise the role of patient-physician communication if preventative measures such as fertility preservation are desired prior to cancer treatments.

The fact that distress was not predicted by objective characteristics of the disease (such as type of diagnosis, stage, or type of treatment) is consistent with evidence from the literature [16] and supports the core premise of the CSM [24–26] that subjective conceptualisation of disease determines one’s response to illness.

The subjective conceptualisation of illness in this study was measured through participants’ illness perceptions. The research focusing on infertility in otherwise healthy women indicates that distress related to the condition is predicted by the perception of more severe consequences of infertility and less control over the condition [43–45]. The findings of this study, however, indicate that although the indicators of cognitive representation (i.e., consequences and identity) contributed to the distress, it was in fact the emotional representation of the illness that best predicted the levels of distress. In other words, the more concerned women were about their illness and the more emotionally burdened they were by cancer diagnosis, the more fertility related-distress they experienced.

Not only the emotional representation of the illness itself, but also what women brought to the situation from the outset, namely their affective predisposition determined the level of post-treatment fertility-related distress. Women who described themselves as generally experiencing more negative emotions also reported higher levels of fertility-related distress. Some research indicates that people who express higher levels of negative affect generally score higher on self-report measures of distress [46]. For this reason, in this study the negative affectivity was used as a control variable. Although it remained significant in the final model predicting distress, so did the emotional representation of illness suggesting that the disease-specific response predicted fertility-related distress above the general negative affectivity understood as one’s predisposition.

Another factor which predicted fertility-related distress was the regret related to the outcome of treatments – the so called ‘outcome regret’ [47]. Women who regretted the fact that cancer treatments impacted their fertility were more likely to experience fertility-related distress than those who did not experience regret. Studies which investigated the concept of fertility-related regret among young women diagnosed with cancer mainly concentrated on the other side of the issue, namely the extent to which women experience regret with respect to the decisions about fertility preservation [16]. The existing evidence suggests that counselling about fertility [48] and provision of decisional aids [49] can minimise regret with respect to decisions about fertility preservation. It does not, however, answer the question about the extent to which regret can impact fertility-related distress. To our knowledge, this study is the first to demonstrate the relationship between treatment-related regret and the increased risk of fertility-related distress post-cancer.

The particular focus of this study was to investigate the role of culture in determining distress related to fertility after cancer. Although the variables reflecting potential cultural differences in the importance attached to fertility (e.g., cultural disapproval of not having children, psychological, social, and utilitarian VOC) did not predict fertility-related distress, the country of origin did: Polish participants experienced more fertility-related distress. This finding can potentially be explained by the differences between Polish and British cultures which were not covered by the culture specific questions included but which nonetheless exist. Polish culture is very family-orientated and attached to traditional values often dictated by the Catholic Church which stresses the importance of having children and condemns contraception and abortion [50]. The prevalence of such beliefs in society might make the situation in which a woman is unable to have children more stressful. As suggested previously by Seknus et al. [22], women from Eastern Europe are generally less likely to accept the risk of infertility related to chemotherapy compared to their counterparts from Western Europe. However, the reasons for that require further research.

While the desire to have children has been widely reported to be a predictor of fertility-related distress, the mechanisms behind this relationship remain unknown. Although additional investigations conducted for the purpose of this study should be treated with caution due to their exploratory nature, they shed some light on this association.

Separate mediation analyses suggest that desire to have children could affect fertility-related distress through its impact on four other variables – the treatment-related regret, psychological VOC, perceived consequences, and emotional burden of the disease. In other words, experiencing a stronger desire to have children before cancer diagnosis was not only directly related to higher levels of fertility-related distress post-cancer but also resulted in higher regret with respect to the treatment outcome, more perceived consequences, and more emotional burden, and through these relationships indirectly affected distress levels. A stronger wish to have children also seemed to determine the degree of importance attached to the psychological rewards related to having children and via this mechanism influenced distress levels.

In a subsequent multiple mediation analysis, all four mediators remained statistically significant. The paths leading through the consequences as well as emotional representation of illness appear to be in line with the CSM, which suggests that factors inherent to self (e.g., desire to have children) can affect illness perception, and these in turn influence the response to illness. This draws attention to the fact that illness perceptions can be influenced not only by the characteristics of the particular disease one suffers from, but also by factors seemingly unrelated to one’s health. The particular contribution of this preliminary finding lies in the fact that while desire to have children is a non-modifiable factor affecting distress, both perceived consequences and emotional representation of illness could potentially be amenable to interventions to tackle fertility-related distress.

The path involving the psychological VOC could potentially be linked to the socio-cultural dimensions of having children with women who desired children more also perceiving their psychological value as more prominent and through this mechanism being affected by higher levels of fertility-related distress.

The socio-cultural influences were explored further through the analysis of the effect that country of origin had on the last significant mediator, namely treatment-related regret and this was done through the means of moderated mediation. The results of this investigation suggest that while for the British participants a higher desire to have children contributed to higher treatment outcome regret and indirectly to more fertility-related distress, this indirect relationship did not exist in the Polish subsample. Among Polish women, the wish to have children, although related to distress, did not have effect on regret. With the lack of cross-cultural research in the field it is difficult to explain this finding, nonetheless, it may be due to the organisational differences between the Polish and British medical systems. While the UK-based National Institute for Health and Care Excellence (NICE) guidelines suggest discussing fertility preservation with all cancer patients [12] and the National Health Service (NHS) has services in place to facilitate fertility preservation for cancer patients, the same is not true for Poland. Therefore, British women who desired children may have regretted not acting upon the possibility of preserving fertility, while at the same time Polish women, not having had that opportunity, did not experience the regret.

More importantly, however, what this finding indicates is that different factors might be playing a role in determining levels of distress across different cultural settings. This stresses the need for cross-cultural research in the field and the importance of physicians’ awareness of cross-cultural differences between their patients.

### Strengths and limitations of the study

The two main strengths of this study are its use of a theoretical framework and its cross-cultural design. The use of the CSM at the level of study design has subsequently allowed us to interpret the findings within its context which contributes to a better understanding of the determinants of fertility-related distress in women with gynecological or breast cancer. This project has also been one of the first cross-cultural studies exploring fertility issues related to cancer, and the first one attempting to uncover how the socio-cultural context may affect fertility-related distress in young female cancer patients.

Despite its strengths, this study also has limitations that need to be accounted for while interpreting its results. At the level of recruitment, it is possible that women who chose to participate in this project were more interested in fertility issues surrounding a cancer diagnosis. This self-selected group may therefore differ from the overall population of young women diagnosed with gynaecological or breast cancer, which limits the generalisability of the findings. At the level of study design, this was a cross-sectional study which means that certain information (e. g., desire to have children before cancer diagnosis) were collected retrospectively. While this allowed us to investigate the associations between the measured variables, it limited our ability of this study to determine the relationships of causality.

## Conclusions

Recent progress in cancer treatment has translated into increased survival rates among cancer patients [1]. Despite this, cancer diagnosis and treatment are not indifferent to the lives of the patients who often struggle with both the emotional and physical consequences of cancer, including, fertility-related distress. This study contributes to the growing field of research focusing on this issue which is commonly faced by young women diagnosed with cancer and adds to its understanding.

First, the results of this study suggest that distress related to fertility follows the assumptions of the CSM in that it seems to be determined by the way one conceptualises one’s illness rather than by objective cancer characteristics and it appears to be contingent on the emotional, rather than cognitive representation of illness. Hence, interventions tackling the process of conceptualising illness among young women could potentially improve their psychosocial outcomes in the survivorship period, and alleviate the levels of distress they experience.

Fertility-related distress was also determined by the desire to have children and treatment-related regret. While the desire to have children is a rather non-modifiable factor, if addressed early through open physician-patient communication, it could guide cancer treatment and fertility preservation decisions which could in turn potentially prevent treatment-related regret. However, as this study suggests there might be cross-cultural differences with respect to fertility-related distress as well as its determinants. Therefore, solutions and preventative measures effective among a particular group of patients might not necessarily apply to a culturally different group. This may prove a challenge to physicians working in multicultural societies, however, more evidence still needs to be gathered.

## Additional files

Additional file 1: **Table S1**. Study inclusion and exclusion criteria. (DOCX 12 kb)

Additional file 2: **Table S2**. Multivariate model predicting total fertility-related distress with consequences (IPQ1) entered in the final block. **Table S3**. Multivariate model predicting total fertility-related distress with timeline (IPQ2) entered in the final block. **Table S4**. Multivariate model predicting total fertility-related distress with identity (IPQ5) entered in the final block. **Table S5**. Multivariate model predicting total fertility-related distress with illness concern (IPQ6) entered in the final block. **Table S6**. Multivariate model predicting total fertility-related distress with illness coherence (IPQ7) entered in the final block. **Table S7**. Multivariate model predicting total fertility-related distress with illness emotional representation (IPQ8) entered in the final block (DOCX 40 kb)

Additional file 3: **Table S8**. Mediation model 1 including desire to have children as predictor, treatment-related regret as mediator and fertility-related distress as outcome. **Figure S1**. Mediation model 1 including desire to have children as predictor, treatment-related regret as mediator and fertility-related distress as outcome (*p ≤ 0.05, **p < 0.01). **Table S9**. Mediation model 2 including desire to have children as predictor, psychological VOC as mediator and fertility-related distress as outcome. **Figure S2**. Mediation model 2 including desire to have children as predictor, psychological VOC as mediator and fertility-related distress as outcome (*p ≤ 0.05, **p < 0.01). **Table 10**. Mediation model 3 with desire to have children as predictor, illness consequences as mediator and fertility-related distress as outcome. **Figure S3**. Mediation model 3 with desire to have children as predictor, illness consequences as mediator and fertility-related distress as outcome (*p ≤ 0.05, **p < 0.01). **Table 11**. Mediation model 4 with desire to have children as predictor, emotional representation as mediator and fertility-related distress as outcome. **Figure S4**. Mediation model 4 with desire to have children as predictor, emotional representation as mediator and fertility-related distress as outcome (*p ≤ 0.05, **p < 0.01). Simple mediation models predicting fertility-related distress. (DOCX 139 kb)

Additional file 4: **Table S12**. Details of multiple mediation model. (DOCX 12 kb)

Additional file 5: **Table S13**. Spearman correlation coefficients for the 'desire to have children', fertility-related distress and the significant mediators for the British subsample of study participants. **Table S14**. Spearman correlation coefficients for the 'desire to have children', fertility-related distress and the significant mediators for the Polish subsample of study participants. **Figure S5**. The conditional effect of desire to have children on treatment-related regret. **Figure S6**. A visual representation of the conditional indirect effect of the desire to have children on fertility-related distress as a function of the country of origin. (DOCX 53 kb)

## Abbreviations

Brief-IPQ: Brief Illness perception questionnaire
CSM: Common Sense Model
IES-R: Impact of Event Scale Revised
NA: Negative affect
NHS: National Health Service
NICE: National Institute for Health and Care Excellence
PANAS: Positive and Negative Affect Scale
SHARE: Scottish Health Research Register
VOC: Value of children
