# Supplementary material for: Determinants of fertility issues experienced by young women diagnosed with breast or gynaecological cancer – a quantitative, cross-cultural study
Source: BMC Cancer. 2018 Sep 6;18:874. doi: 10.1186/s12885-018-4766-y (PMC6127915; doi:10.1186/s12885-018-4766-y)
Supplement: Supplementary file 1 — Table S1. Study inclusion and exclusion criteria. (DOCX 12 kb) [file 12885_2018_4766_MOESM1_ESM.docx]

Table S1 Study inclusion and exclusion criteria

| Gynaecological or breast cancer |
| --- |
| Diagnosed between the ages of 18-45 years old |
| Menstruating at the time of diagnosis |
| Had chemotherapy (neo-adjuvant or adjuvant) as part of their treatment if they were diagnosed with breast cancer |
| Finished active treatment (with the exception of endocrine therapy for breast cancer) prior to participation |
| Had no known evidence of cancer recurrence at the time of participation |
| Spoke English or Polish |
