# Supplementary material for: Determinants of fertility issues experienced by young women diagnosed with breast or gynaecological cancer – a quantitative, cross-cultural study
Source: BMC Cancer. 2018 Sep 6;18:874. doi: 10.1186/s12885-018-4766-y (PMC6127915; doi:10.1186/s12885-018-4766-y)
Supplement: Supplementary file 2 — Table S2. Multivariate model predicting total fertility-related distress with consequences (IPQ1) entered in the final block. Table S3. Multivariate model predicting total fertility-related distress with timeline (IPQ2) entered in the final block. Table S4. Multivariate model predicting total fertility-related distress with identity (IPQ5) entered in the final block. Table S5. Multivariate model predicting total fertility-related distress with illness concern (IPQ6) entered in the final block. Table S6. Multivariate model predicting total fertility-related distress with illness coherence (IPQ7) entered in the final block. Table S7. Multivariate model predicting total fertility-related distress with illness emotional representation (IPQ8) entered in the final block (DOCX 40 kb) [file 12885_2018_4766_MOESM2_ESM.docx]

Table S2. Multivariate model predicting total fertility-related distress with consequences (IPQ1) entered in the final block

|  | **B** | **SE B** | **β** | ***p*** |
| --- | --- | --- | --- | --- |
| **Step 1 – control variables** | | | | |
| Constant | 25.49 | 10.37 |  | *≤0.05* |
| Age at diagnosis | -0.45 | 0.25 | -0.14 | *n.s.* |
| Country of origin (Britain vs Poland) | 12.45 | 3.67 | 0.24 | *<0.01* |
| Type of cancer (gynaecological vs breast) | 3.14 | 5.01 | 0.06 | *n.s.* |
| Type of treatment (sterile vs uncertain fertility) | -9.62 | 4.16 | -0.22 | *≤0.05* |
| Recruitment site (other vs online) | -10.42 | 3.20 | -0.24 | *<0.01* |
| Childbearing status (no vs yes) | -3.24 | 2.90 | -0.08 | *n.s.* |
| Negative affect | 1.06 | 0.15 | 0.46 | *<0.01* |
| **Step 2 – desire to have children** | | | | |
| Constant | 3.61 | 11.71 |  | *n.s.* |
| Age at diagnosis | -0.17 | 0.25 | -0.05 | *n.s.* |
| Country of origin (Britain vs Poland) | 13.28 | 3.53 | 0.27 | *<0.01* |
| Type of cancer (gynaecological vs breast) | 2.32 | 4.82 | 0.04 | *n.s.* |
| Type of treatment (sterile vs uncertain fertility) | -8.18 | 4.01 | -0.18 | *≤0.05* |
| Recruitment site (other vs online) | -9.27 | 3.09 | -0.21 | *<0.01* |
| Childbearing status (no vs yes) | -0.48 | 2.89 | -0.01 | *n.s.* |
| Negative affect | 1.06 | 0.15 | 0.46 | *<0.01* |
| Desire to have children | 3.43 | 0.97 | 0.25 | *<0.01* |
| **Step 3 – treatment-related regret** | | | | |
| Constant | -0.41 | 11.49 |  | *n.s.* |
| Age at diagnosis | -0.14 | 0.24 | -0.04 | *n.s.* |
| Country of origin (Britain vs Poland) | 13.82 | 3.44 | 0.28 | *<0.01* |
| Type of cancer (gynaecological vs breast) | 1.45 | 4.70 | 0.03 | *n.s.* |
| Type of treatment (sterile vs uncertain fertility) | -5.76 | 4.00 | -013 | *n.s.* |
| Recruitment site (other vs online) | -8.18 | 3.03 | -0.19 | *<0.01* |
| Childbearing status (no vs yes) | 0.63 | 2.84 | 0.02 | *n.s.* |
| Negative affect | 1.03 | 0.14 | 0.45 | *<0.01* |
| Desire to have children | 2.97 | 0.96 | 0.22 | *<0.01* |
| Treatment related regret (no vs all others) | 8.65 | 3.00 | 0.19 | *<0.01* |
| **Step 4 – culture-related variables** | | | | |
| Constant | -6.25 | 11.80 |  | *n.s.* |
| Age at diagnosis | -0.22 | 0.25 | -0.07 | *n.s.* |
| Country of origin (Britain vs Poland) | 12.21 | 3.67 | 0.25 | *<0.01* |
| Type of cancer (gynaecological vs breast) | 2.21 | 4.71 | 0.04 | *n.s.* |
| Type of treatment (sterile vs uncertain fertility) | -7.26 | 4.07 | -0.16 | *n.s.* |
| Recruitment site (other vs online) | -8.31 | 3.05 | -0.19 | *<0.01* |
| Childbearing status (no vs yes) | -0.80 | 3.07 | -0.02 | *n.s.* |
| Negative affect | 0.94 | 0.15 | 0.41 | *<0.01* |
| Desire to have children | 2.30 | 1.02 | 0.17 | *≤0.05* |
| Treatment related regret (no vs all others) | 8.27 | 3.05 | 0.18 | *<0.01* |
| Cultural disapproval of childlessness (no vs all others) | 3.85 | 2.74 | 0.09 | *n.s.* |
| Value of children – utilitarian | 1.50 | 2.66 | 0.05 | *n.s.* |
| Value of children – social | -0.99 | 3.01 | -0.03 | *n.s.* |
| Value of children – psychological | 3.28 | 2.26 | 0.12 | *n.s.* |
| **Step 5 – illness perceptions (consequences)** | | | | |
| Constant | -8.49 | 11.70 |  | *n.s.* |
| Age at diagnosis | -0.22 | 0.24 | -0.07 | *n.s.* |
| Country of origin (Britain vs Poland) | 11.33 | 3.65 | 0.23 | *<0.01* |
| Type of cancer (gynaecological vs breast) | 1.48 | 4.66 | 0.03 | *n.s.* |
| Type of treatment (sterile vs uncertain fertility) | -6.20 | 4.05 | -0.14 | *n.s.* |
| Recruitment site (other vs online) | -7.62 | 3.03 | -0.18 | *≤0.05* |
| Childbearing status (no vs yes) | -1.73 | 3.07 | -0.04 | *n.s.* |
| Negative affect | 0.81 | 0.16 | 0.35 | *<0.01* |
| Desire to have children | 1.99 | 1.01 | 0.15 | *n.s.* |
| Treatment related regret (no vs all others) | 7.43 | 3.04 | 0.16 | *≤0.05* |
| Cultural disapproval of childlessness (no vs all others) | 3.48 | 2.71 | 0.08 | *n.s.* |
| Value of children – utilitarian | 1.40 | 2.63 | 0.05 | *n.s.* |
| Value of children – social | -0.88 | 2.97 | -0.03 | *n.s.* |
| Value of children – psychological | 3.59 | 2.24 | 0.13 | *n.s.* |
| Consequences (IPQ1) | 1.14 | 0.55 | 0.14 | *≤0.05* |
| *Note.* Step 1: *R*^2^ = 0.466, adjusted *R*^2^ = 0.438, *F*(7, 134) = 16.69, *p* < 0.01 Step 2: *R*^2^ = 0.512, adjusted *R*^2^ = 0.482, *F*(1, 133) = 12.55, *p* < 0.01, Δ *R*^2^ = 0.046 Step 3: *R*^2^ = 0.541, adjusted *R*^2^ = 0.509, *F*(1, 132) = 8.31, *p* < 0.01, Δ *R*^2^ = 0.029 Step 4: *R*^2^ = 0.561, adjusted *R*^2^ = 0.516, *F*(4, 128) = 1.48, *p* = n.s., Δ *R*^2^ = 0.020 Step 5: *R*^2^ = 0.575, adjusted *R*^2^ = 0.529, *F*(1, 127) = 4.29, *p* ≤ 0.05, Δ *R*^2^ =0.014 The assumptions of the model have been tested and achieved. Sample size of 164 was deemed adequate to include 14 predictors in the analysis (Green, 1991). Collinearity tolerance statistics and variance inflation factors were within acceptable ranges (>0.1, <10 respectively). The Durbin-Watson statistic for the final model was 2.17 (acceptable range 1-3), suggesting that residuals were independent. The standardised residual P-P and scatter plots were inspected visually and indicated that the assumptions of normality and homoscedasticity were met. | | | | |

Table S3. Multivariate model predicting total fertility-related distress with timeline (IPQ2) entered in the final block

|  | **B** | **SE B** | **β** | **p** |
| --- | --- | --- | --- | --- |
| **Step 1 – control variables** | | | | |
| Constant | 25.49 | 10.37 |  | *≤0.05* |
| Age at diagnosis | -0.45 | 0.25 | -0.14 | *n.s.* |
| Country of origin (Britain vs Poland) | 12.45 | 3.67 | 0.24 | *<0.01* |
| Type of cancer (gynaecological vs breast) | 3.14 | 5.01 | 0.06 | *n.s.* |
| Type of treatment (sterile vs uncertain fertility) | -9.62 | 4.16 | -0.22 | *≤0.05* |
| Recruitment site (other vs online) | -10.42 | 3.20 | -0.24 | *<0.01* |
| Childbearing status (no vs yes) | -3.24 | 2.90 | -0.08 | *n.s.* |
| Negative affect | 1.06 | 0.15 | 0.46 | *<0.01* |
| **Step 2 – desire to have children** | | | | |
| Constant | 3.61 | 11.71 |  | *n.s.* |
| Age at diagnosis | -0.17 | 0.25 | -0.05 | *n.s.* |
| Country of origin (Britain vs Poland) | 13.28 | 3.53 | 0.27 | *<0.01* |
| Type of cancer (gynaecological vs breast) | 2.32 | 4.82 | 0.04 | *n.s.* |
| Type of treatment (sterile vs uncertain fertility) | -8.18 | 4.01 | -0.18 | *≤0.05* |
| Recruitment site (other vs online) | -9.27 | 3.09 | -0.21 | *<0.01* |
| Childbearing status (no vs yes) | -0.48 | 2.89 | -0.01 | *n.s.* |
| Negative affect | 1.06 | 0.15 | 0.46 | *<0.01* |
| Desire to have children | 3.43 | 0.97 | 0.25 | *<0.01* |
| **Step 3 – treatment-related regret** | | | | |
| Constant | -0.41 | 11.49 |  | *n.s.* |
| Age at diagnosis | -0.14 | 0.24 | -0.04 | *n.s.* |
| Country of origin (Britain vs Poland) | 13.82 | 3.44 | 0.28 | *<0.01* |
| Type of cancer (gynaecological vs breast) | 1.45 | 4.70 | 0.03 | *n.s.* |
| Type of treatment (sterile vs uncertain fertility) | -5.76 | 4.00 | -0.13 | *n.s.* |
| Recruitment site (other vs online) | -8.18 | 3.03 | -0.19 | *<0.01* |
| Childbearing status (no vs yes) | 0.63 | 2.84 | 0.02 | *n.s.* |
| Negative affect | 1.03 | 0.14 | 0.45 | *<0.01* |
| Desire to have children | 2.97 | 0.96 | 0.22 | *<0.01* |
| Treatment related regret (no vs all others) | 8.65 | 3.00 | 0.19 | *<0.01* |
| **Step 4 – culture-related variables** |  |  |  |  |
| Constant | -6.25 | 11.80 |  | *n.s.* |
| Age at diagnosis | -0.22 | 0.25 | -0.07 | *n.s.* |
| Country of origin (Britain vs Poland) | 12.21 | 3.67 | 0.25 | *<0.01* |
| Type of cancer (gynaecological vs breast) | 2.21 | 4.71 | 0.04 | *n.s.* |
| Type of treatment (sterile vs uncertain fertility) | -7.26 | 4.07 | -0.16 | *n.s.* |
| Recruitment site (other vs online) | -8.31 | 3.05 | -0.19 | *<0.01* |
| Childbearing status (no vs yes) | -0.80 | 3.07 | -0.02 | *n.s.* |
| Negative affect | 0.94 | 0.15 | 0.41 | *<0.01* |
| Desire to have children | 2.30 | 1.02 | 0.17 | *≤0.05* |
| Treatment related regret (no vs all others) | 8.27 | 3.05 | 0.18 | *<0.01* |
| Cultural disapproval of childlessness (no vs all others) | 3.85 | 2.74 | 0.09 | *n.s.* |
| Value of children – utilitarian | 1.50 | 2.66 | 0.05 | *n.s.* |
| Value of children – social | -0.99 | 3.01 | -0.03 | *n.s.* |
| Value of children – psychological | 3.28 | 2.26 | 0.12 | *n.s.* |
| **Step 5 – illness perceptions (timeline)** |  |  |  |  |
| Constant | -5.97 | 11.76 |  | *n.s.* |
| Age at diagnosis | -0.26 | 0.25 | -0.08 | *n.s.* |
| Country of origin (Britain vs Poland) | 10.99 | 3.76 | 0.23 | *<0.01* |
| Type of cancer (gynaecological vs breast) | 1.21 | 4.75 | 0.02 | *n.s.* |
| Type of treatment (sterile vs uncertain fertility) | -6.88 | 4.07 | -0.16 | *n.s.* |
| Recruitment site (other vs online) | -8.11 | 3.04 | -0.18 | *<0.01* |
| Childbearing status (no vs yes) | -0.91 | 3.06 | -0.02 | *n.s.* |
| Negative affect | 0.89 | 0.15 | 0.39 | *<0.01* |
| Desire to have children | 2.26 | 1.01 | 0.16 | *≤0.05* |
| Treatment related regret (no vs all others) | 7.99 | 3.05 | 0.17 | *≤0.05* |
| Cultural disapproval of childlessness (no vs all others) | 3.76 | 2.73 | 0.09 | *n.s.* |
| Value of children – utilitarian | 1.73 | 2.66 | 0.06 | *n.s.* |
| Value of children – social | -1.0 | 3.00 | -0.03 | *n.s.* |
| Value of children – psychological | 3.32 | 2.25 | 0.12 | *n.s.* |
| Timeline (IPQ2) | 0.49 | 0.36 | 0.09 | *n.s.* |
| *Note.* Step 1: *R*^2^ = 0.466, adjusted *R*^2^ = 0.438, *F*(7, 134) = 16.69, *p* < 0.01 Step 2: *R*^2^ = 0.512, adjusted *R*^2^ = 0.482, *F*(1, 133) = 12.55, *p* < 0.01, Δ *R* ^2^= 0.046 Step 3: *R*^2^ = 0.541, adjusted *R*^2^ = 0.509, *F*(1, 132) = 8.31, *p* < 0.01, Δ *R*^2^ = 0.029 Step 4: *R*^2^ = 0.561, adjusted *R*^2^ = 0.516, *F*(4, 128) = 1.48, *p* = n.s., Δ *R*^2^ = 0.020 Step 5: *R*^2^ = 0.567, adjusted *R*^2^ = 0.520, *F*(1, 127) = 1.85, *p* = n.s., Δ *R*^2^ = 0.006 The assumptions of the model have been tested and acheved. Sample size of 164 was deemed adequate to include 14 predictors in the analysis (Green, 1991). Collinearity tolerance statistics and variance inflation factors were within acceptable ranges (>0.1, <10 respectively). The Durbin-Watson statistic for the final model was 2.27 (acceptable range 1-3), suggesting that residuals were independent. The standardised residual P-P and scatter plots were inspected visually and indicated that the assumptions of normality and homoscedasticity were met. | | | | |

Table S4. Multivariate model predicting total fertility-related distress with identity (IPQ5) entered in the final block

|  | **B** | **SE B** | **β** | ***p*** |
| --- | --- | --- | --- | --- |
| **Step 1 – control variables** | | | | |
| Constant | 25.49 | 10.37 |  | *≤0.05* |
| Age at diagnosis | -0.45 | 0.25 | -0.14 | *n.s.* |
| Country of origin (Britain vs Poland) | 12.45 | 3.67 | 0.24 | *<0.01* |
| Type of cancer (gynaecological vs breast) | 3.14 | 5.01 | 0.06 | *n.s.* |
| Type of treatment (sterile vs uncertain fertility) | -9.62 | 4.16 | -0.22 | *≤0.05* |
| Recruitment site (other vs online) | -10.42 | 3.20 | -0.24 | *<0.01* |
| Childbearing status (no vs yes) | -3.24 | 2.90 | -0.08 | *n.s.* |
| Negative affect | 1.06 | 0.15 | 0.46 | *<0.01* |
| **Step 2 – desire to have children** | | | | |
| Constant | 3.61 | 11.71 |  | *n.s.* |
| Age at diagnosis | -0.17 | 0.25 | -0.05 | *n.s.* |
| Country of origin (Britain vs Poland) | 13.28 | 3.53 | 0.27 | *<0.01* |
| Type of cancer (gynaecological vs breast) | 2.32 | 4.82 | 0.04 | *n.s.* |
| Type of treatment (sterile vs uncertain fertility) | -8.18 | 4.01 | -0.18 | *≤0.05* |
| Recruitment site (other vs online) | -9.27 | 3.09 | -0.21 | *<0.01* |
| Childbearing status (no vs yes) | -0.48 | 2.89 | -0.01 | *n.s.* |
| Negative affect | 1.06 | 0.15 | 0.46 | *<0.01* |
| Desire to have children | 3.43 | 0.97 | 0.25 | *<0.01* |
| **Step 3 – treatment-related regret** | | | | |
| Constant | -0.41 | 11.49 |  | *n.s.* |
| Age at diagnosis | -0.14 | 0.24 | -0.04 | *n.s.* |
| Country of origin (Britain vs Poland) | 13.82 | 3.44 | 0.28 | *<0.01* |
| Type of cancer (gynaecological vs breast) | 1.45 | 4.70 | 0.03 | *n.s.* |
| Type of treatment (sterile vs uncertain fertility) | -5.76 | 4.00 | -0.13 | *n.s.* |
| Recruitment site (other vs online) | -8.18 | 3.03 | -0.19 | *<0.01* |
| Childbearing status (no vs yes) | 0.63 | 2.84 | 0.02 | *n.s.* |
| Negative affect | 1.03 | 0.14 | 0.45 | *<0.01* |
| Desire to have children | 2.97 | 0.96 | 0.22 | *<0.01* |
| Treatment related regret (no vs all others) | 8.65 | 3.00 | 0.19 | *<0.01* |
| **Step 4 – culture-related variables** |  |  |  |  |
| Constant | -6.25 | 11.80 |  | *n.s.* |
| Age at diagnosis | -0.22 | 0.25 | -0.07 | *n.s.* |
| Country of origin (Britain vs Poland) | 12.21 | 3.67 | 0.25 | *<0.01* |
| Type of cancer (gynaecological vs breast) | 2.21 | 4.71 | 0.04 | *n.s.* |
| Type of treatment (sterile vs uncertain fertility) | -7.26 | 4.07 | -0.16 | *n.s.* |
| Recruitment site (other vs online) | -8.31 | 3.05 | -0.19 | *<0.01* |
| Childbearing status (no vs yes) | -0.80 | 3.07 | -0.01 | *n.s.* |
| Negative affect | 0.94 | 0.15 | 0.41 | *<0.01* |
| Desire to have children | 2.30 | 1.02 | 0.17 | *≤0.05* |
| Treatment related regret (no vs all others) | 8.27 | 3.05 | 0.18 | *<0.01* |
| Cultural disapproval of childlessness (no vs all others) | 3.85 | 2.74 | 0.09 | *n.s.* |
| Value of children – utilitarian | 1.50 | 2.66 | 0.05 | *n.s.* |
| Value of children – social | -1.0 | 3.01 | -0.03 | *n.s.* |
| Value of children – psychological | 3.28 | 2.26 | 0.12 | *n.s.* |
| **Step 5 – illness perceptions (consequences)** |  |  |  |  |
| Constant | -8.01 | 11.60 |  | *n.s.* |
| Age at diagnosis | -0.22 | 0.24 | -0.07 | *n.s.* |
| Country of origin (Britain vs Poland) | 11.56 | 3.61 | 0.24 | *<0.01* |
| Type of cancer (gynaecological vs breast) | 0.15 | 4.69 | 0.003 | *n.s.* |
| Type of treatment (sterile vs uncertain fertility) | -5.63 | 4.05 | -0.13 | *n.s.* |
| Recruitment site (other vs online) | -7.45 | 3.01 | -0.17 | *≤0.05* |
| Childbearing status (no vs yes) | -2.89 | 3.13 | -0.07 | *n.s.* |
| Negative affect | 0.85 | 0.15 | 0.37 | *<0.01* |
| Desire to have children | 1.98 | 1.01 | 0.14 | *n.s.* |
| Treatment related regret (no vs all others) | 7.93 | 3.00 | 0.17 | *<0.01* |
| Cultural disapproval of childlessness (no vs all others) | 3.56 | 2.69 | 0.08 | *n.s.* |
| Value of children – utilitarian | 1.61 | 2.61 | 0.06 | *n.s.* |
| Value of children – social | -1.68 | 2.97 | -0.06 | *n.s.* |
| Value of children – psychological | 4.05 | 2.24 | 0.15 | *n.s.* |
| Identity (IPQ5) | 1.20 | 0.49 | 0.16 | *≤0.05* |
| *Note.* Step 1: *R*^2^ = 0.466, adjusted *R*^2^ = 0.438, *F*(7, 134) = 16.69, *p* < 0.01 Step 2: *R*^2^ = 0.512, adjusted *R*^2^ = 0.482, *F*(1, 133) = 12.55, *p* < 0.01, Δ *R*^2^ = 0.046 Step 3: *R*^2^ = 0.541, adjusted *R*^2^ = 0.509, *F*(1, 132) = 8.31, *p* < 0.05, Δ *R*^2^ = 0.029 Step 4: *R*^2^ = 0.561, adjusted *R*^2^ = 0.516, *F*(4, 128) = 1.48, *p* = n.s., Δ *R*^2^ = 0.020 Step 5: *R*^2^ = 0.581, adjusted *R*^2^ = 0.535, *F*(1, 127) = 6.04, *p ≤ 0.05*, Δ *R* ^2^= 0.020 The assumptions of the model have been tested and achieved. Sample size of 164 was deemed adequate to include 14 predictors in the analysis (Green, 1991). Collinearity tolerance statistics and variance inflation factors were within acceptable ranges (>0.1, <10 respectively). The Durbin-Watson statistic for the final model was 2.18 (acceptable range 1-3), suggesting that residuals were independent. The standardised residual P-P and scatter plots were inspected visually and indicated that the assumptions of normality and homoscedasticity were met. | | | | |

Table S5. Multivariate model predicting total fertility-related distress with illness concern (IPQ6) entered in the final block

|  | **B** | **SE B** | **β** | ***p*** |
| --- | --- | --- | --- | --- |
| **Step 1 – control variables** | | | | |
| Constant | 25.49 | 10.37 |  | *≤0.05* |
| Age at diagnosis | -0.45 | 0.25 | -0.14 | *n.s.* |
| Country of origin (Britain vs Poland) | 12.45 | 3.67 | 0.24 | *<0.01* |
| Type of cancer (gynaecological vs breast) | 3.14 | 5.01 | 0.06 | *n.s.* |
| Type of treatment (sterile vs uncertain fertility) | -9.62 | 4.16 | -0.22 | *≤0.05* |
| Recruitment site (other vs online) | -10.42 | 3.20 | -0.24 | *<0.01* |
| Childbearing status (no vs yes) | -3.24 | 2.90 | -0.08 | *n.s.* |
| Negative affect | 1.06 | 0.153 | 0.46 | *<0.01* |
| **Step 2 – desire to have children** | | | | |
| Constant | 3.61 | 11.71 |  | *n.s.* |
| Age at diagnosis | -0.17 | 0.25 | -0.05 | *n.s.* |
| Country of origin (Britain vs Poland) | 13.28 | 3.53 | 0.27 | *<0.01* |
| Type of cancer (gynaecological vs breast) | 2.32 | 4.82 | 0.04 | *n.s.* |
| Type of treatment (sterile vs uncertain fertility) | -8.18 | 4.01 | -0.18 | *≤0.05* |
| Recruitment site (other vs online) | -9.27 | 3.09 | -0.21 | *<0.01* |
| Childbearing status (no vs yes) | -0.48 | 2.89 | -0.01 | *n.s.* |
| Negative affect | 1.06 | 0.15 | 0.46 | *<0.01* |
| Desire to have children | 3.43 | 0.97 | 0.25 | *<0.01* |
| **Step 3 – treatment-related regret** | | | | |
| Constant | -0.41 | 11.49 |  | *n.s.* |
| Age at diagnosis | -0.14 | 0.24 | -0.04 | *n.s.* |
| Country of origin (Britain vs Poland) | 13.82 | 3.44 | 0.28 | *<0.01* |
| Type of cancer (gynaecological vs breast) | 1.45 | 4.70 | 0.03 | *n.s.* |
| Type of treatment (sterile vs uncertain fertility) | -5.76 | 4.00 | -0.13 | *n.s.* |
| Recruitment site (other vs online) | -8.18 | 3.03 | -0.19 | *<0.01* |
| Childbearing status (no vs yes) | 0.63 | 2.84 | 0.02 | *n.s.* |
| Negative affect | 1.03 | 0.14 | 0.45 | *<0.01* |
| Desire to have children | 2.97 | 0.96 | 0.22 | *<0.01* |
| Treatment related regret (no vs all others) | 8.65 | 3.00 | 0.19 | *<0.01* |
| **Step 4 – culture-related variables** |  |  |  |  |
| Constant | -6.25 | 11.80 |  | *n.s.* |
| Age at diagnosis | -0.22 | 0.25 | -0.07 | *n.s.* |
| Country of origin (Britain vs Poland) | 12.21 | 3.67 | 0.25 | *<0.01* |
| Type of cancer (gynaecological vs breast) | 2.21 | 4.71 | 0.04 | *n.s.* |
| Type of treatment (sterile vs uncertain fertility) | -7.26 | 4.07 | -0.16 | *n.s.* |
| Recruitment site (other vs online) | -8.31 | 3.05 | -0.19 | *<0.01* |
| Childbearing status (no vs yes) | -0.80 | 3.07 | -0.02 | *n.s.* |
| Negative affect | 0.94 | 0.15 | 0.41 | *<0.01* |
| Desire to have children | 2.30 | 1.02 | 0.17 | *≤0.05* |
| Treatment related regret (no vs all others) | 8.27 | 3.05 | 0.18 | *<0.01* |
| Cultural disapproval of childlessness (no vs all others) | 3.85 | 2.74 | 0.09 | *n.s.* |
| Value of children – utilitarian | 1.50 | 2.66 | 0.05 | *n.s.* |
| Value of children – social | -1.00 | 3.01 | -0.03 | *n.s.* |
| Value of children – psychological | 3.28 | 2.26 | 0.12 | *n.s.* |
| **Step 5 – illness perceptions (consequences)** |  |  |  |  |
| Constant | -14.83 | 11.64 |  | *n.s.* |
| Age at diagnosis | -0.18 | 0.24 | -0.06 | *n.s.* |
| Country of origin (Britain vs Poland) | 13.29 | 3.54 | 0.27 | *<0.01* |
| Type of cancer (gynaecological vs breast) | 0.91 | 4.54 | 0.02 | *n.s.* |
| Type of treatment (sterile vs uncertain fertility) | -5.79 | 3.94 | -0.13 | *n.s.* |
| Recruitment site (other vs online) | -5.67 | 3.04 | -0.13 | *n.s.* |
| Childbearing status (no vs yes) | -2.02 | 2.98 | -0.05 | *n.s.* |
| Negative affect | 0.72 | 0.16 | 0.32 | *<0.01* |
| Desire to have children | 2.20 | 0.98 | 0.16 | *≤0.05* |
| Treatment related regret (no vs all others) | 8.63 | 2.94 | 0.19 | *<0.01* |
| Cultural disapproval of childlessness (no vs all others) | 3.84 | 2.63 | 0.09 | *n.s.* |
| Value of children – utilitarian | 1.21 | 2.56 | 0.04 | *n.s.* |
| Value of children – social | -1.54 | 2.90 | -0.05 | *n.s.* |
| Value of children – psychological | 4.22 | 2.19 | 0.15 | *n.s.* |
| Illness concern (IPQ6) | 1.63 | 0.49 | 0.23 | *<0.01* |
| *Note.* Step 1: *R*^2^ = 0.466, adjusted *R*^2^ = 0.438, *F*(7, 134) = 16.69, *p* < 0.01 Step 2: *R*^2^ = 0.512, adjusted *R*^2^ = 0.482, *F*(1, 133) = 12.55, *p* < 0.01, Δ *R*^2^ = 0.046 Step 3: *R*^2^ = 0.541, adjusted *R*^2^ = 0.509, *F*(1, 132) = 8.31, *p* < 0.01, Δ *R*^2^ = 0.029 Step 4: *R*^2^ = 0.561, adjusted *R*^2^ = 0.516, *F*(4, 128) = 1.48, *p* = n.s., Δ *R*^2^ = 0.020 Step 5: *R*^2^ = 0.597, adjusted *R*^2^ = 0.552, *F*(1, 127) = 11.26, *p* < 0.01, Δ *R*^2^ = 0.036 The assumptions of the model have been tested and achieved. Sample size of 164 was deemed adequate to include 14 predictors in the analysis (Green, 1991). Collinearity tolerance statistics and variance inflation factors were within acceptable ranges (>0.1, <10 respectively). The Durbin-Watson statistic for the final model was 2.29 (acceptable range 1-3), suggesting that residuals were independent. The standardised residual P-P and scatter plots were inspected visually and indicated that the assumptions of normality and homoscedasticity were met. | | | | |

Table S6. Multivariate model predicting total fertility-related distress with illness coherence (IPQ7) entered in the final block

|  | **B** | **SE B** | **β** | ***p*** |
| --- | --- | --- | --- | --- |
| **Step 1 – control variables** | | | | |
| Constant | 25.49 | 10.37 |  | *≤0.05* |
| Age at diagnosis | -0.45 | 0.25 | -0.14 | *n.s.* |
| Country of origin (Britain vs Poland) | 12.45 | 3.67 | 0.24 | *<0.01* |
| Type of cancer (gynaecological vs breast) | 3.14 | 5.01 | 0.06 | *n.s.* |
| Type of treatment (sterile vs uncertain fertility) | -9.62 | 4.16 | -0.22 | *≤0.05* |
| Recruitment site (other vs online) | -10.42 | 3.20 | -0.24 | *<0.01* |
| Childbearing status (no vs yes) | -3.24 | 2.90 | -0.08 | *n.s.* |
| Negative affect | 1.06 | 0.153 | 0.46 | *<0.01* |
| **Step 2 – desire to have children** | | | | |
| Constant | 3.61 | 11.71 |  | *n.s.* |
| Age at diagnosis | -0.17 | 0.25 | -0.05 | *n.s.* |
| Country of origin (Britain vs Poland) | 13.28 | 3.53 | 0.27 | *<0.01* |
| Type of cancer (gynaecological vs breast) | 2.32 | 4.82 | 0.04 | *n.s.* |
| Type of treatment (sterile vs uncertain fertility) | -8.18 | 4.01 | -0.18 | *≤0.05* |
| Recruitment site (other vs online) | -9.27 | 3.09 | -0.21 | *<0.01* |
| Childbearing status (no vs yes) | -0.48 | 2.89 | -0.01 | *n.s.* |
| Negative affect | 1.06 | 0.15 | 0.46 | *<0.01* |
| Desire to have children | 3.43 | 0.97 | 0.25 | *<0.01* |
| **Step 3 – treatment-related regret** | | | | |
| Constant | -0.41 | 11.49 |  | *n.s.* |
| Age at diagnosis | -0.14 | 0.24 | -0.04 | *n.s.* |
| Country of origin (Britain vs Poland) | 13.82 | 3.44 | 0.28 | *<0.01* |
| Type of cancer (gynaecological vs breast) | 1.45 | 4.70 | 0.03 | *n.s.* |
| Type of treatment (sterile vs uncertain fertility) | -5.76 | 4.00 | -0.13 | *n.s.* |
| Recruitment site (other vs online) | -8.18 | 3.03 | -0.19 | *<0.01* |
| Childbearing status (no vs yes) | 0.63 | 2.84 | 0.02 | *n.s.* |
| Negative affect | 1.03 | 0.14 | 0.45 | *<0.01* |
| Desire to have children | 2.97 | 0.96 | 0.22 | *<0.01* |
| Treatment related regret (no vs all others) | 8.65 | 3.00 | 0.19 | *<0.01* |
| **Step 4 – culture-related variables** | | | | |
| Constant | -6.25 | 11.80 |  | *n.s.* |
| Age at diagnosis | -0.22 | 0.25 | -0.07 | *n.s.* |
| Country of origin (Britain vs Poland) | 12.21 | 3.67 | 0.25 | *<0.01* |
| Type of cancer (gynaecological vs breast) | 2.21 | 4.71 | 0.04 | *n.s.* |
| Type of treatment (sterile vs uncertain fertility) | -7.26 | 4.07 | -0.16 | *n.s.* |
| Recruitment site (other vs online) | -8.31 | 3.05 | -0.19 | *<0.01* |
| Childbearing status (no vs yes) | -0.80 | 3.07 | -0.02 | *n.s.* |
| Negative affect | 0.94 | 0.15 | 0.41 | *<0.01* |
| Desire to have children | 2.30 | 1.02 | 0.17 | *≤0.05* |
| Treatment related regret (no vs all others) | 8.27 | 3.05 | 0.18 | *<0.01* |
| Cultural disapproval of childlessness (no vs all others) | 3.85 | 2.74 | 0.09 | *n.s.* |
| Value of children – utilitarian | 1.50 | 2.66 | 0.05 | *n.s.* |
| Value of children – social | -1.00 | 3.01 | -0.03 | *n.s.* |
| Value of children – psychological | 3.28 | 2.26 | 0.12 | *n.s.* |
| **Step 5 – illness perceptions (consequences)** | | | | |
| Constant | 1.24 | 12.82 |  | *n.s.* |
| Age at diagnosis | -0.22 | 0.25 | -0.07 | *n.s.* |
| Country of origin (Britain vs Poland) | 11.37 | 3.70 | 0.23 | *<0.01* |
| Type of cancer (gynaecological vs breast) | 2.29 | 4.69 | 0.04 | *n.s.* |
| Type of treatment (sterile vs uncertain fertility) | -7.68 | 4.07 | -0.17 | *n.s.* |
| Recruitment site (other vs online) | -8.43 | 3.04 | -0.19 | *<0.01* |
| Childbearing status (no vs yes) | -0.70 | 3.06 | -0.02 | *n.s.* |
| Negative affect | 0.92 | 0.15 | 0.40 | *<0.01* |
| Desire to have children | 2.61 | 1.03 | 0.19 | *≤0.05* |
| Treatment related regret (no vs all others) | 7.66 | 3.07 | 0.17 | *≤0.05* |
| Cultural disapproval of childlessness (no vs all others) | 3.08 | 2.78 | 0.07 | *n.s.* |
| Value of children – utilitarian | 1.05 | 2.67 | 0.04 | *n.s.* |
| Value of children – social | -0.87 | 3.00 | -0.03 | *n.s.* |
| Value of children – psychological | 3.24 | 2.25 | 0.12 | *n.s.* |
| Coherence (IPQ7) | -0.80 | 0.55 | -0.10 | *n.s.* |
| *Note.* Step 1: *R*^2^ = 0.466, adjusted *R*^2^ = 0.438, *F*(7, 134) = 16.69, *p* < 0.01 Step 2: *R*^2^ = 0.512, adjusted *R*^2^ = 0.482, *F*(1, 133) = 12.55, *p* < 0.01, Δ *R*^2^ = 0.046 Step 3: *R*^2^ = 0.541, adjusted *R*^2^ = 0.509, *F*(1, 132) = 8.31, *p* < 0.01, Δ *R*^2^ = 0.029 Step 4: *R*^2^ = 0.561, adjusted *R*^2^ = 0.516, *F*(4, 128) = 1.48, *p* = n.s., Δ *R*^2^ = 0.020 Step 5: *R*^2^ = 0.568, adjusted *R*^2^ = 0.521, *F*(1, 127) = 2.14, *p* = n.s., Δ *R*^2^ = 0.007 The assumptions of the model have been tested and achieved. Sample size of 164 was deemed adequate to include 14 predictors in the analysis (Green, 1991). Collinearity tolerance statistics and variance inflation factors were within acceptable ranges (>0.1, <10 respectively). The Durbin-Watson statistic for the final model was 2.29 (acceptable range 1-3), suggesting that residuals were independent. The standardised residual P-P and scatter plots were inspected visually and indicated that the assumptions of normality and homoscedasticity were met. | | | | |

Table S7. Multivariate model predicting total fertility-related distress with illness emotional representation (IPQ8) entered in the final block

|  | **B** | **SE B** | **β** | ***p*** |
| --- | --- | --- | --- | --- |
| **Step 1 – control variables** | | | | |
| Constant | 25.49 | 10.37 |  | *≤0.05* |
| Age at diagnosis | -0.45 | 0.25 | -0.14 | *n.s.* |
| Country of origin (Britain vs Poland) | 12.45 | 3.67 | 0.24 | *<0.01* |
| Type of cancer (gynaecological vs breast) | 3.14 | 5.01 | 0.06 | *n.s.* |
| Type of treatment (sterile vs uncertain fertility) | -9.62 | 4.16 | -0.22 | *≤0.05* |
| Recruitment site (other vs online) | -10.42 | 3.20 | -0.24 | *<0.01* |
| Childbearing status (no vs yes) | -3.24 | 2.90 | -0.08 | *n.s.* |
| Negative affect | 1.06 | 0.153 | 0.46 | *<0.01* |
| **Step 2 – desire to have children** | | | | |
| Constant | 3.61 | 11.71 |  | *n.s.* |
| Age at diagnosis | -0.17 | 0.25 | -0.05 | *n.s.* |
| Country of origin (Britain vs Poland) | 13.28 | 3.53 | 0.27 | *<0.01* |
| Type of cancer (gynaecological vs breast) | 2.32 | 4.82 | 0.04 | *n.s.* |
| Type of treatment (sterile vs uncertain fertility) | -8.18 | 4.01 | -0.18 | *≤0.05* |
| Recruitment site (other vs online) | -9.27 | 3.09 | -0.21 | *<0.01* |
| Childbearing status (no vs yes) | -0.48 | 2.89 | -0.01 | *n.s.* |
| Negative affect | 1.06 | 0.15 | 0.46 | *<0.01* |
| Desire to have children | 3.43 | 0.97 | 0.25 | *<0.01* |
| **Step 3 – treatment-related regret** | | | | |
| Constant | -0.41 | 11.49 |  | *n.s.* |
| Age at diagnosis | -0.14 | 0.24 | -0.044 | *n.s.* |
| Country of origin (Britain vs Poland) | 13.82 | 3.44 | 0.28 | *<0.01* |
| Type of cancer (gynaecological vs breast) | 1.45 | 4.70 | 0.03 | *n.s.* |
| Type of treatment (sterile vs uncertain fertility) | -5.76 | 4.00 | -0.13 | *n.s.* |
| Recruitment site (other vs online) | -8.18 | 3.03 | -0.19 | *<0.01* |
| Childbearing status (no vs yes) | 0.63 | 2.84 | 0.02 | *n.s.* |
| Negative affect | 1.03 | 0.14 | 0.45 | *<0.01* |
| Desire to have children | 2.97 | 0.96 | 0.22 | *<0.01* |
| Treatment related regret (no vs all others) | 8.65 | 3.00 | 0.19 | *<0.01* |
| **Step 4 – culture-related variables** | | | | |
| Constant | -6.25 | 11.80 |  | *n.s.* |
| Age at diagnosis | -0.22 | 0.25 | -0.07 | *n.s.* |
| Country of origin (Britain vs Poland) | 12.21 | 3.67 | 0.25 | *<0.01* |
| Type of cancer (gynaecological vs breast) | 2.21 | 4.71 | 0.04 | *n.s.* |
| Type of treatment (sterile vs uncertain fertility) | -7.26 | 4.07 | -0.16 | *n.s.* |
| Recruitment site (other vs online) | -8.31 | 3.05 | -0.19 | *<0.01* |
| Childbearing status (no vs yes) | -0.80 | 3.07 | -0.02 | *n.s.* |
| Negative affect | 0.94 | 0.15 | 0.41 | *<0.01* |
| Desire to have children | 2.30 | 1.02 | 0.17 | *≤0.05* |
| Treatment related regret (no vs all others) | 8.27 | 3.05 | 0.18 | *<0.01* |
| Cultural disapproval of childlessness (no vs all others) | 3.85 | 2.74 | 0.09 | *n.s.* |
| Value of children – utilitarian | 1.50 | 2.66 | 0.05 | *n.s.* |
| Value of children – social | -1.00 | 3.01 | -0.03 | *n.s.* |
| Value of children – psychological | 3.28 | 2.26 | 0.12 | *n.s.* |
| **Step 5 – illness perceptions (consequences)** | | | | |
| Constant | -18.42 | 11.83 |  | *n.s.* |
| Age at diagnosis | -0.08 | 0.24 | -0.02 | *n.s.* |
| Country of origin (Britain vs Poland) | 14.48 | 3.57 | 0.30 | *<0.01* |
| Type of cancer (gynaecological vs breast) | 0.93 | 4.52 | 0.02 | *n.s.* |
| Type of treatment (sterile vs uncertain fertility) | -5.25 | 3.95 | -0.12 | *n.s.* |
| Recruitment site (other vs online) | -6.36 | 2.97 | -0.15 | *≤0.05* |
| Childbearing status (no vs yes) | -2.71 | 2.99 | -0.06 | *n.s.* |
| Negative affect | 0.64 | 0.17 | 0.28 | *<0.01* |
| Desire to have children | 1.99 | 0.98 | 0.15 | *≤0.05* |
| Treatment related regret (no vs all others) | 7.89 | 2.93 | 0.17 | *<0.01* |
| Cultural disapproval of childlessness (no vs all others) | 3.67 | 2.62 | 0.08 | *n.s.* |
| Value of children – utilitarian | 0.77 | 2.56 | 0.03 | *n.s.* |
| Value of children – social | -1.80 | 2.90 | -0.06 | *n.s.* |
| Value of children – psychological | 4.54 | 2.20 | 0.16 | *≤0.05* |
| Emotional representation (IPQ8) | 2.07 | 0.59 | 0.26 | *<0.01* |
| *Note.* Step 1: *R*^2^ = 0.466, adjusted *R*^2^ = 0.438, *F*(7, 134) = 16.69, *p* < 0.01 Step 2: *R*^2^ = 0.512, adjusted *R*^2^ = 0.482, *F*(1, 133) = 12.55, *p* < 0.01, Δ *R*^2^ = 0.046 Step 3: *R*^2^ = 0.541, adjusted *R*^2^ = 0.509, *F*(1, 132) = 8.31, *p* < 0.01, Δ *R*^2^ = 0.029 Step 4: *R*^2^ = 0.561, adjusted *R*^2^ = 0.516, *F*(4, 128) = 1.48, *p* = n.s., Δ *R*^2^ = 0.020 Step 5: *R*^2^ = 0.600, adjusted *R*^2^ = 0.556, *F*(1, 127) = 12.363, *p* < 0.01, Δ *R*^2^ = 0.039 The assumptions of the model have been tested and achieved. Sample size of 164 was deemed adequate to include 14 predictors in the analysis (Green, 1991). Collinearity tolerance statistics and variance inflation factors were within acceptable ranges (>0.1, <10 respectively). The Durbin-Watson statistic for the final model was 2.22 (acceptable range 1-3), suggesting that residuals were independent. The standardised residual P-P and scatter plots were inspected visually and indicated that the assumptions of normality and homoscedasticity were met. | | | | |
