# Supplementary material for: Determinants of fertility issues experienced by young women diagnosed with breast or gynaecological cancer – a quantitative, cross-cultural study
Source: BMC Cancer. 2018 Sep 6;18:874. doi: 10.1186/s12885-018-4766-y (PMC6127915; doi:10.1186/s12885-018-4766-y)
Supplement: Supplementary file 3 — Table S8. Mediation model 1 including desire to have children as predictor, treatment-related regret as mediator and fertility-related distress as outcome. Figure S1. Mediation model 1 including desire to have children as predictor, treatment-related regret as mediator and fertility-related distress as outcome (*p ≤ 0.05, **p < 0.01). Table S9. Mediation model 2 including desire to have children as predictor, psychological VOC as mediator and fertility-related distress as outcome. Figure S2. Mediation model 2 including desire to have children as predictor, psychological VOC as mediator and fertility-related distress as outcome (*p ≤ 0.05, **p < 0.01). Table 10. Mediation model 3 with desire to have children as predictor, illness consequences as mediator and fertility-related distress as outcome. Figure S3. Mediation model 3 with desire to have children as predictor, illness consequences as mediator and fertility-related distress as outcome (*p ≤ 0.05, **p < 0.01). Table 11. Mediation model 4 with desire to have children as predictor, emotional representation as mediator and fertility-related distress as outcome. Figure S4. Mediation model 4 with desire to have children as predictor, emotional representation as mediator and fertility-related distress as outcome (*p ≤ 0.05, **p < 0.01). Simple mediation models predicting fertility-related distress. (DOCX 139 kb) [file 12885_2018_4766_MOESM3_ESM.docx]

Table S8. Mediation model 1 including desire to have children as predictor, treatment-related regret as mediator and fertility-related distress as outcome

|  |  | **Consequent** | | | | | | |
| --- | --- | --- | --- | --- | --- | --- | --- | --- |
|  |  | **Treatment-related regret** | | |  | **Fertility-related distress** | | |
| **Antecedent** |  | B | SE | *p* |  | B | SE | *p* |
| **Constant** | i | 1.03 | 0.15 | *<0.01* | i | 11.09 | 3.71 | *<0.01* |
| **Desire to have children** | a | 0.22 | 0.06 | *<0.01* | c’ | 3.76 | 1.10 | *<0.01* |
| **Treatment related regret** |  | - | - | - | b | 4.97 | 1.58 | *<0.01* |
|  |  | *R*^2^ = 0.10  *F*(1, 151) = 14.69, *p < 0.01* | | |  | *R*^2^ = 0.18  *F*(2, 150) = 16.94, *p < 0.01* | | |


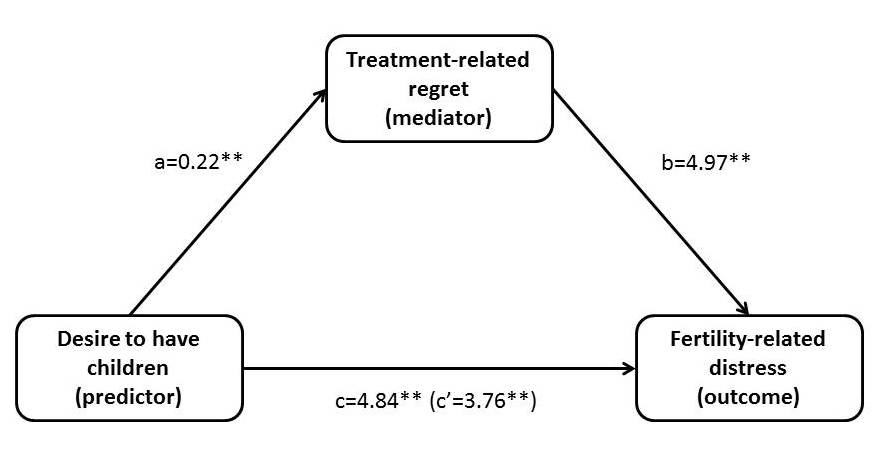


Figure S1. Mediation model 1 including desire to have children as predictor, treatment-related regret as mediator and fertility-related distress as outcome
(**p* ≤ 0.05, ***p* < 0.01)

Table S9. Mediation model 2 including desire to have children as predictor, psychological VOC as mediator and fertility-related distress as outcome

|  |  | **Consequent** | | | | | | |
| --- | --- | --- | --- | --- | --- | --- | --- | --- |
|  |  | **VOC_P** | | |  | **Fertility-related distress** | | |
| **Antecedent** |  | B | SE | *p* |  | B | SE | *p* |
| **Constant** | i | 3.04 | 0.15 | *<0.01* | i | 1.14 | 8.82 | *n.s.* |
| **Desire to have children** | a | 0.12 | 0.04 | *<0.01* | c’ | 4.22 | 1.02 | *<0.01* |
| **VOC_P** |  | - | - | - | b | 4.97 | 2.45 | *≤0.05* |
|  |  | *R*^2^ = 0.06  *F*(1, 151) = 9.06, *p<0.01* | | |  | *R*^2^ = 0.15  *F*(2, 150)12.12, *p<0.01* | | |


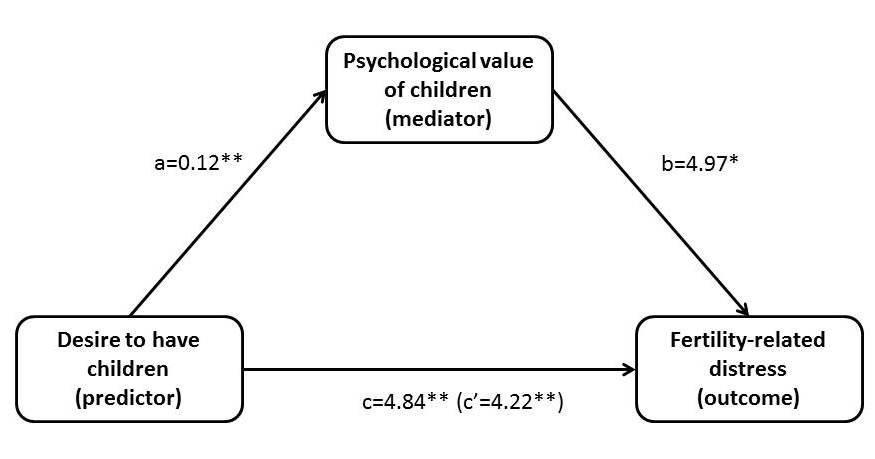


Figure S2. Mediation model 2 including desire to have children as predictor, psychological VOC as mediator and fertility-related distress as outcome (**p* ≤ 0.05, ***p* < 0.01)

Table 10. Mediation model 3 with desire to have children as predictor, illness consequences as mediator and fertility-related distress as outcome

|  |  | **Consequent** | | | | | | |
| --- | --- | --- | --- | --- | --- | --- | --- | --- |
|  |  | **Consequences (IPQ1)** | | |  | **Fertility-related distress** | | |
| **Antecedent** |  | B | SE | *p* |  | B | SE | *p* |
| **Constant** | i | 4.73 | 0.61 | *<0.01* | i | 0.28 | 3.47 | *n.s.* |
| **Desire to have children** | a | 0.29 | 0.14 | *≤0.05* | c’ | 3.98 | 1.00 | *<0.01* |
| **Consequences (IPQ1)** |  | - | - | - | b | 3.29 | 0.58 | *<0.01* |
|  |  | *R*^2^ = 0.03  *F*(1, 152) = 4.15, *p≤0.05* | | |  | *R*^2^ = 0.29  *F*(2, 151) = 44.34, *p<0.01* | | |


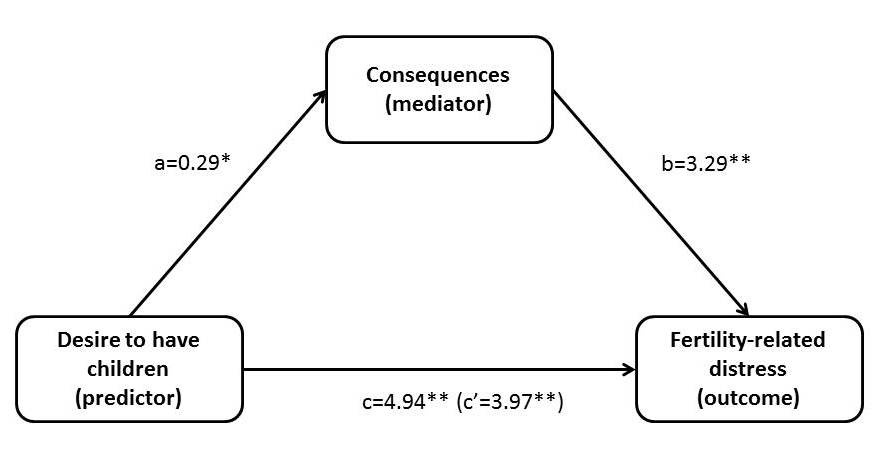


Figure S3. Mediation model 3 with desire to have children as predictor, illness consequences as mediator and fertility-related distress as outcome
(**p* ≤ 0.05, ***p* < 0.01)

Table 11. Mediation model 4 with desire to have children as predictor, emotional representation as mediator and fertility-related distress as outcome

|  |  | **Consequent** | | | | | | |
| --- | --- | --- | --- | --- | --- | --- | --- | --- |
|  |  | **Emotional representation (IPQ8)** | | |  | **Fertility-related distress** | | |
| **Antecedent** |  | B | SE | *p* |  | B | SE | *p* |
| **Constant** | i | 5.72 | 0.48 | *<0.01* | i | -5.46 | 3.66 | *n.s.* |
| **Desire to have children** | a | 0.29 | 0.14 | *≤0.05* | c’ | 3.88 | 0.93 | *<0.01* |
| **Emotional representation (IPQ8)** |  | - | - | - | b | 3.71 | 0.53 | *<0.01* |
|  |  | *R*^2^ = 0.03  *F*(1, 151) = 4.43, *p≤0.05* | | |  | *R*^2^ = 0.34  *F*(2, 150) = 49.62, *p<0.01* | | |


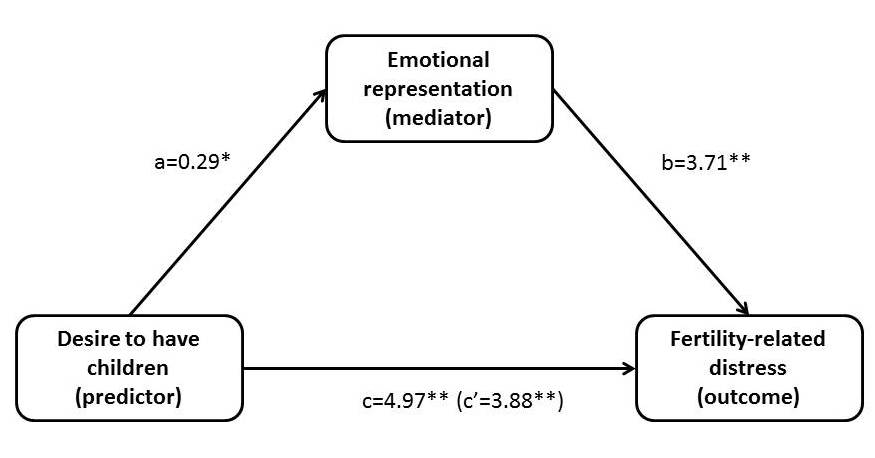


Figure S4. Mediation model 4 with desire to have children as predictor, emotional representation as mediator and fertility-related distress as outcome
(**p* ≤ 0.05, ***p* < 0.01)
