# Supplementary material for: Determinants of fertility issues experienced by young women diagnosed with breast or gynaecological cancer – a quantitative, cross-cultural study
Source: BMC Cancer. 2018 Sep 6;18:874. doi: 10.1186/s12885-018-4766-y (PMC6127915; doi:10.1186/s12885-018-4766-y)
Supplement: Supplementary file 4 — Table S12. Details of multiple mediation model. (DOCX 12 kb) [file 12885_2018_4766_MOESM4_ESM.docx]

Table S12. Details of multiple mediation model

| **Variable** | **Path** | **Unstandardised indirect effect (95% CI)** | **Standardised direct effect (95% CI)** | **% total effect of predictor on outcome through mediator** |
| --- | --- | --- | --- | --- |
| **Treatment-related regret** | a_1_b_1_ | 0.74 (0.21, 1.54) | 0.05 (0.02, 0.11) | 15.43 |
| **Psychological VOC** | a_2_b_2_ | 0.53 (0.07, 1.25) | 0.04 (0.004, 0.09) | 11.14 |
| **Consequences** | a_3_b_3_ | 0.38 (0.009, 1.31) | 0.03 (0.0002, 0.09) | 8.03 |
| **Emotional representation** | a_4_b_4_ | 0.77 (0.12, 1.68) | 0.06 (0.01, 0.12) | 16.07 |
