# Supplementary material for: Determinants of fertility issues experienced by young women diagnosed with breast or gynaecological cancer – a quantitative, cross-cultural study
Source: BMC Cancer. 2018 Sep 6;18:874. doi: 10.1186/s12885-018-4766-y (PMC6127915; doi:10.1186/s12885-018-4766-y)
Supplement: Supplementary file 5 — Table S13. Spearman correlation coefficients for the 'desire to have children', fertility-related distress and the significant mediators for the British subsample of study participants. Table S14. Spearman correlation coefficients for the 'desire to have children', fertility-related distress and the significant mediators for the Polish subsample of study participants. Figure S5. The conditional effect of desire to have children on treatment-related regret. Figure S6. A visual representation of the conditional indirect effect of the desire to have children on fertility-related distress as a function of the country of origin. (DOCX 53 kb) [file 12885_2018_4766_MOESM5_ESM.docx]

Table S13. Spearman correlation coefficients for the 'desire to have children', fertility-related distress and the significant mediators for the British subsample of study participants

| **Variable** |  | **Fertility-related distress** | **Desire to have children** |
| --- | --- | --- | --- |
| **Fertility-related distress** | ρ | 1 | 0.42 |
|  | *p* | *.* | *<0.01* |
| **Desire to have children** | ρ | 0.42 | 1 |
|  | *p* | *<0.01* | *.* |
| **Treatment-related regret** | ρ | 0.48 | 0.40 |
|  | *p* | *<0.01* | *<0.01* |
| **Psychological VOC** | ρ | 0.20 | 0.12 |
|  | *p* | *≤0.05* | *n.s.* |
| **Consequences (IPQ1)** | ρ | 0.47 | 0.16 |
|  | *p* | *<0.01* | *n.s.* |
| **Emotional representation (IPQ8)** | ρ | 0.52 | 0.12 |
|  | *p* | *<0.01* | *n.s.* |
| *Note.* ρ – Spearman’s rho; *p* – significance level | | | |

Table S14. Spearman correlation coefficients for the 'desire to have children', fertility-related distress and the significant mediators for the Polish subsample of study participants

| **Variable** |  | **Fertility-related distress** | **Desire to have children** |
| --- | --- | --- | --- |
| **Fertility-related distress** | ρ | 1 | 0.38 |
|  | *p* | *.* | *≤0.05* |
| **Desire to have children** | ρ | 0.38 | 1 |
|  | *p* | *≤0.05* | *.* |
| **Treatment-related regret** | ρ | 0.12 | 0.03 |
|  | *p* | *n.s.* | *n.s.* |
| **Psychological VOC** | ρ | 0.37 | 0.22 |
|  | *p* | *≤0.05* | *n.s.* |
| **Consequences (IPQ1)** | ρ | 0.47 | 0.15 |
|  | *p* | *≤0.05* | *n.s.* |
| **Emotional representation (IPQ8)** | ρ | 0.67 | 0.22 |
|  | *p* | *<0.01* | *n.s.* |
| *Note.* ρ – Spearman’s rho; *p* – significance level | | | |


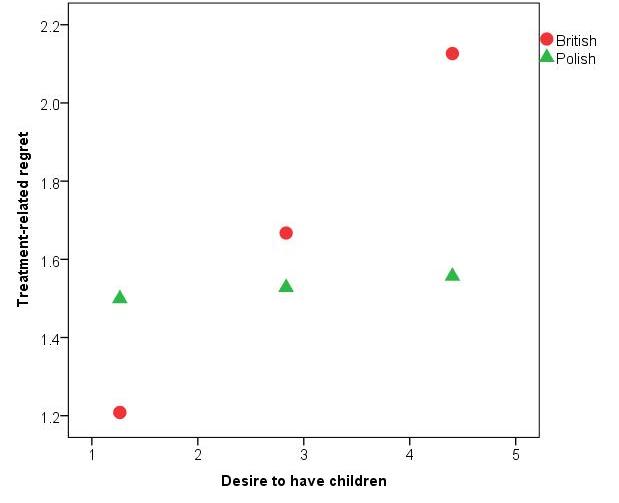


Figure S5. The conditional effect of desire to have children on treatment-related regret


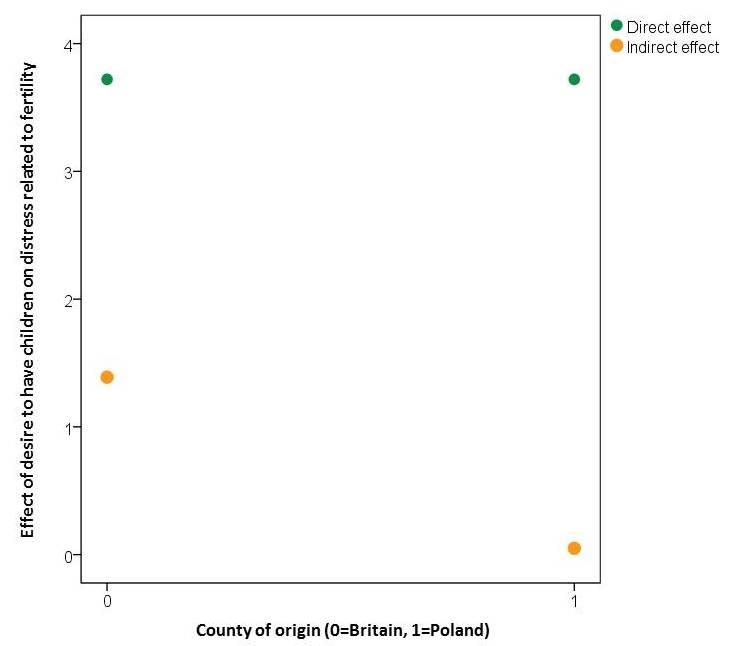


Figure S6. A visual representation of the conditional indirect effect of the desire to have children on fertility-related distress as a function of the country of origin
